# Constructing a Human Atrial Fibre Atlas

**DOI:** 10.1007/s10439-020-02525-w

**Published:** 2020-05-26

**Authors:** Caroline H. Roney, Rokas Bendikas, Farhad Pashakhanloo, Cesare Corrado, Edward J. Vigmond, Elliot R. McVeigh, Natalia A. Trayanova, Steven A. Niederer

**Affiliations:** 1 School of Biomedical Engineering and Imaging Sciences, King’s College London, London, UK; 2 Cardiovascular Division, Department of Medicine, Beth Israel Deaconess Medical Center and Harvard Medical School, Boston, USA; 3 IHU Liryc, Electrophysiology and Heart Modeling Institute, fondation Bordeaux Université, 33600 Pessac, Bordeaux, France; 4 Department of Bioengineering, UC San Diego School of Engineering, San Diego, USA; 5 Department of Biomedical Engineering, Johns Hopkins University, Baltimore, USA; 6 University of Bordeaux, IMB UMR 5251, 33400 Talence, France

**Keywords:** Atrial fibres, Anisotropy, Atrial activation, Atrial fibrillation

## Abstract

Atrial anisotropy affects electrical propagation patterns, anchor locations of atrial reentrant drivers, and atrial mechanics. However, patient-specific atrial fibre fields and anisotropy measurements are not currently available, and consequently assigning fibre fields to atrial models is challenging. We aimed to construct an atrial fibre atlas from a high-resolution DTMRI dataset that optimally reproduces electrophysiology simulation predictions corresponding to patient-specific fibre fields, and to develop a methodology for automatically assigning fibres to patient-specific anatomies. We extended an atrial coordinate system to map the pulmonary veins, vena cava and appendages to standardised positions in the coordinate system corresponding to the average location across the anatomies. We then expressed each fibre field in this atrial coordinate system and calculated an average fibre field. To assess the effects of fibre field on patient-specific modelling predictions, we calculated paced activation time maps and electrical driver locations during AF. In total, 756 activation time maps were calculated (7 anatomies with 9 fibre maps and 2 pacing locations, for the endocardial, epicardial and bilayer surface models of the LA and RA). Patient-specific fibre fields had a relatively small effect on average paced activation maps (range of mean local activation time difference for LA fields: 2.67–3.60 ms, and for RA fields: 2.29–3.44 ms), but had a larger effect on maximum LAT differences (range for LA 12.7–16.6%; range for RA 11.9–15.0%). A total of 126 phase singularity density maps were calculated (7 anatomies with 9 fibre maps for the LA and RA bilayer models). The fibre field corresponding to anatomy 1 had the highest median PS density map correlation coefficient for LA bilayer simulations (0.44 compared to the other correlations, ranging from 0.14 to 0.39), while the average fibre field had the highest correlation for the RA bilayer simulations (0.61 compared to the other correlations, ranging from 0.37 to 0.56). For sinus rhythm simulations, average activation time is robust to fibre field direction; however, maximum differences can still be significant. Patient specific fibres are more important for arrhythmia simulations, particularly in the left atrium. We propose using the fibre field corresponding to DTMRI dataset 1 for LA simulations, and the average fibre field for RA simulations as these optimally predicted arrhythmia properties.

**Electronic supplementary material:**

The online version of this article (doi:10.1007/s10439-020-02525-w) contains supplementary material, which is available to authorized users.

## Introduction

Atrial myocytes are arranged with a preferential myocyte orientation that varies both across the atrial surface and through the atrial wall.^17^ The angle of this preferential myocyte orientation is often referred to as the fibre orientation. In contrast to the ventricles that have a regular transmural fibre orientation,^15^,^26^ the fibres and anatomy in the atria exhibit higher inter-individual variability.^16^,^17^ Pashakhanloo *et al*. performed high resolution *ex vivo* diffusion tensor magnetic resonance imaging of eight human atria to demonstrate that there are features of the atrial anatomy that are consistent across subjects—including the septopulmonary bundle, the crista terminalis and the pectinate muscle fibres—but the exact location and orientation of atrial bundles varies.^29^ Atrial fibre direction within the pulmonary veins (PV) and around the junction of the PV with the left atrial body are particularly variable between subjects; with reported fibre arrangements including spiralling, circular, chaotic and longitudinal fibres, with either similar or different fibres on the endocardial and epicardial surfaces.^16^,^18^,^42^.

Structural anisotropy affects electrical propagation patterns^22^ locations of atrial reentrant drivers,^34^ and atrial mechanics.^19^ However, current imaging modalities are not suitable for global *in vivo* measurements of atrial fibre directions;^48^ as such it is challenging to personalise atrial fibre distributions for patient specific assessment or computational models. There are multiple methodologies for including atrial fibres in either 2D surface or 3D volumetric computational meshes. These include rule-based techniques to capture histological descriptions of atrial anatomy and fibre structure.^12^,^20^,^24^,^25^,^44^ An alternative approach that may be used to incorporate fibre direction in computational models is to register a fibre atlas to a patient specific geometry.^20^,^28^,^36^ Fibre atlases used in current modelling pipelines are either synthetic, constructed in a rule-based manner based on histological and morphological descriptions of atrial tissue, or are derived from single large animal datasets.^48^ These approaches, however, exhibit limited validation, are not representative of individual patients, are not necessarily from self consistent data sets, and do not always cover both left and right atria. In addition, there is growing interest within the cardiac electrophysiology community in variability, and these deterministic rule-based methods do not allow direct propagation of uncertainty through to model simulation output.

To address the need for a self consistent data driven representative fibre map and the corresponding uncertainty quantification, we develop a biatrial fibre atlas constructed as an average of the high-resolution DTMRI dataset of Pashakhanloo *et al*.^29^ We first project the fibres for each of the endocardial and epicardial left and right atrial surfaces onto a local coordinate system. We then construct mean left and right atrial endocardial and epicardial fibre fields and evaluate the local standard deviation of the fibre orientations. We compare simulated local activation time maps for each of the meshes with each of the individual fibre fields or the mean field. We also simulate atrial fibrillation (AF) across the different combinations of anatomy and fibre field and compare arrhythmia properties to the patient specific fibre field simulations. Finally, we use the activation time maps and arrhythmia simulation results to determine which of the fibre fields is most representative of the patient-specific fibre fields, and to propose a fibre atlas for personalising fibres in patient specific models.

## Materials and Methods

**Figure 1:**
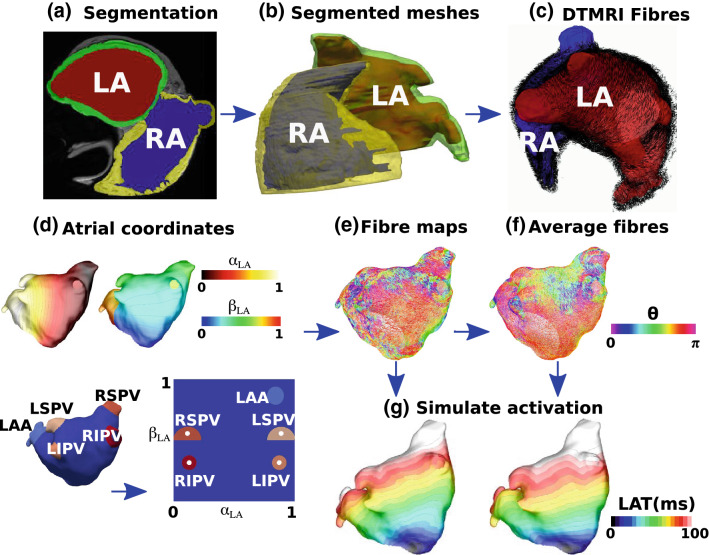
Atlas from DTMRI data. (a) MRI data were segmented to give the LA blood pool (red), the LA wall (green), the RA blood pool (blue) and the RA wall (yellow). (b) The left and right endocardial and epicardial surfaces were meshed. (c) DTMRI fibres were extracted for each of the LA and RA. (d) Atrial coordinates were calculated (shown here for the LA) and used to express each atrium in a 2D coordinate system. (e) Fibres were expressed in the UAC basis, with a UAC fibre angle. (f) Fibre fields from each of the left or right atrial endocardial or epicardial shells were registered to a common shell using UAC and an average fibre field was calculated. (g) Simulations were run to evaluate the effects of fibre field on activation time maps (examples shown here correspond to the fibre fields in (e) and (f).

We initially describe the segmentation and meshing steps used to construct left and right atrial endocardial and epicardial meshes with fibre fields for each of the *ex-vivo *DTMRI datasets (“Data Modalities” and “Segmentation, Meshing and Fibre Assignment” sections). We further develop our atrial coordinate system to fix the locations of the pulmonary veins, vena cava and appendages at positions in the coordinate system corresponding to the average location across the anatomies to minimise the average distortion (“Atrial Coordinates” section). We use the atrial coordinate system to transfer fibre fields between atrial geometries and register all fibres to a single mesh to calculate a mean fibre field with uncertainty (“Fibre Mapping and Calculating Mean Fibre Fields” section), which is visualised using a streamline based approach (“Fibre Visualisation” section). Finally we describe the methodologies used for simulating local activation time maps ("Local Activation Time Simulations" section) and arrhythmia simulations (“Arrhythmia Simulations” section). The main steps in this methodology are shown in the schematic in Fig. 1. Data corresponding to the endocardial and epicardial surface meshes, their fibre fields and the average fibre fields are available online (10.5281/zenodo.3764917 and 10.18742/RDM01-596).

### Data Modalities

All DTMRI data used here are from the study of Pashakhanloo *et al*. in which human hearts were procured through the National Disease Research Interchange (Philadelphia, PA).^29^ We used seven out of eight biatrial datasets from Pashakhanloo *et al*.^29^ because for one of the atria we were unable to achieve a segmentation that could be used for calculation of the universal atrial coordinates used for registration. Biatrial anatomies were obtained from non-diffusion weighted images (b0), together with DTMRI images reconstructed to $0.4\,\text {mm}\times 0.4\,\text {mm}\times 0.4\,\text {mm}$ for assigning fibre fields to the atrial meshes.

### Segmentation, Meshing and Fibre Assignment

To construct an endocardial surface of the left atria, we segmented the blood pool of the b0 images using our open source segmentation platform, the CEMRGapp (www.cemrgapp.com),^39^ which was developed using the medical imaging toolkit (MITK).^46^ Specifically, we used the region growing tool on individual slices, together with MITK 3D interpolation. Segmentations were then manually corrected. Right atrial blood pool segmentations required the use of additional tools to accurately segment the detailed pectinate muscle structures. As such, we used 3D region growing with linear thresholding and a connectivity filter of 26-connectivity for each voxel. To outline the pectinate muscle structure, we used a Canny edge detection filter. To segment the left or right atrial epicardial surfaces, we initially combined the corresponding endocardial segmentation with the left or right atrial components of the masked image. We then used an 8-connectivity filter, together with a cluster size tolerance of 300 pixels, to fill any holes in the combined segmentation. Specifically, identified clusters bounded by the blood pool were categorised as blood pool, whereas clusters surrounded by mask pixels were assumed to be holes in the mask. Finally, the segmentation was manually edited using ITK-snap^47^ software to remove any erroneous voxels or fill any missing areas.

Simulation meshes were constructed from the segmented images using the following sequence of steps. First, a marching cubes algorithm implemented within the medical image registration toolkit (https://github.com/BioMedIA/MIRTK) was used to extract an isosurface from the segmentation. This surface was then modified in Meshlab^7^ using the following sequence of filters to create a watertight surface: screened Poisson surface reconstruction, marching cubes, and quadric edge collapse decimation. The resulting surface was then trimmed to open the mesh at the pulmonary veins (PV), superior vena cava (SVC), inferior vena cava (IVC), coronary sinus (CS) and mitral and tricuspid valves (MV and TV) using Paraview software.^1^ Finally, this mesh was remeshed using mmgtools meshing software (https://github.com/MmgTools/mmg) to produce meshes suitable for simulations, with an average edge length of 0.34 mm.

Fibres were assigned to each element of these surface meshes by finding the closest fibre in the DTMRI fibre field to the element mid-point, and projecting this fibre to the surface.

### Atrial Coordinates

To register data across atrial anatomies, to create an average fibre atlas and to calculate streamlines for fibre visualisation, each of the left and right atrial endocardial surfaces was expressed in the universal atrial coordinate (UAC) system.^33^ UAC were calculated using two Laplace solves for each atria with Dirichlet boundary conditions of zero and one applied along two sets of boundary nodes. For the LA, the two coordinates were chosen as a septal to lateral coordinate ($\alpha_{\text{LA}}$), and a coordinate from the posterior mitral valve, over the roof, to the anterior mitral valve ($\beta_{\text{LA}}$), since these fields are close to orthogonal and use easily identifiable atrial structures. Equivalently for the RA, the first coordinate passes from the lateral tricuspid valve to the septal tricuspid valve ($\alpha_{\text{RA}}$), and the second coordinate was from the inferior vena cava to the superior vena cava ($\beta_{\text{RA}}$).

**Figure 2:**
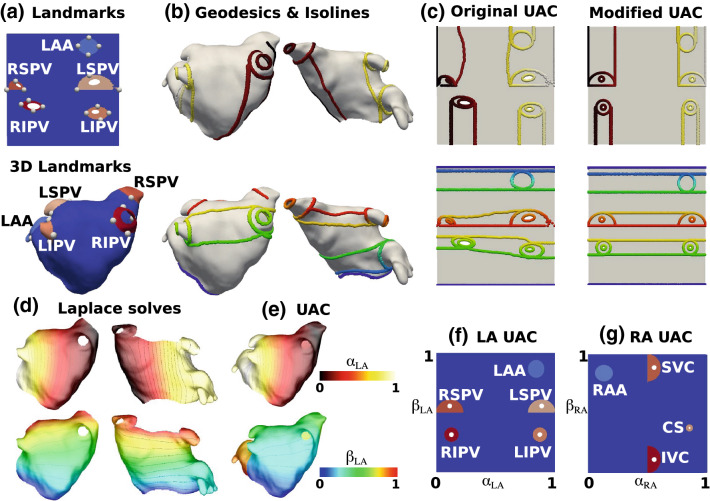
UAC landmarks and paths: (a) 2D points were automatically selected in the original UAC system and mapped to 3D. (b) Geodesic paths or isolines were calculated between the selected 3D points and assigned boundary values, indicated by colour (top: lateral-septal coordinate ($\alpha _{\text{LA}}$), bottom posterior-anterior coordinate ($\beta _{\text{LA}}$)). These paths were chosen to fix the location of the PV and LAA as explained in the Supplementary material Section 1.1. (c) These boundary paths are shown in the original UAC (left) and modified UAC (right) system. (d) Laplace solves were performed for the posterior (left) and anterior (right) meshes separately subject to the boundary conditions, for the $\alpha _{\text{LA}}$ (top) and $\beta _{\text{LA}}$ (bottom) coordinates. (e) The posterior and anterior mesh solutions were combined to give UAC for the full mesh. (f) Final LA 2D UAC representation. (g) Equivalent steps were used to give the final RA 2D UAC representation

Atrial fibres are inherently defined relative to anatomical structures (the PV, SVC, IVC, CS, left and right atrial appendages). Consequently, mapping fibres between cases requires that these structures have the same UAC values on all meshes. To create a coordinate system with anatomical structures located at the same UAC, the original UAC system was extended to include additional boundary conditions. Detailed methodology on mapping anatomical structures to atlas coordinate locations, the choice of boundary conditions and the Laplace solves used to construct the UAC system are given in the supplementary material, and shown in Fig. 2.

This implementation uses the VTK and VMTK^2^ python libraries, together with carpentry software for the Laplace solves (https://carp.medunigraz.at/carputils/). Visualisation was performed using either Paraview^1^ or meshalyzer software (https://github.com/cardiosolv/meshalyzer).

### Fibre Mapping and Calculating Mean Fibre Fields

**Figure 3:**
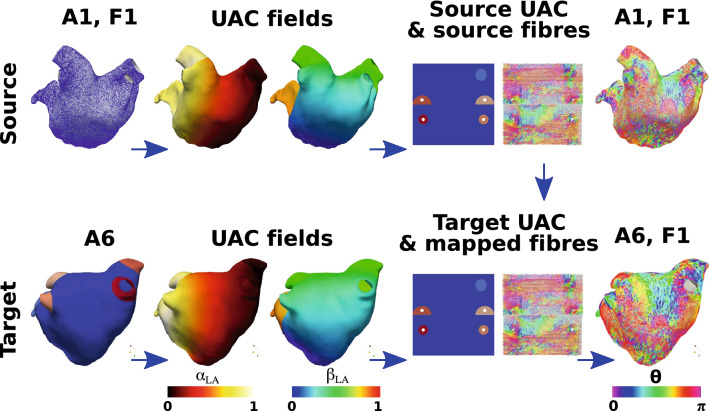
UAC fibre mapping. An example is shown for mapping LA anatomy 1 with its associated fibre field (the *source*, top row) to anatomy 6 (the *target*, bottom row). Both the source and target anatomies are expressed in UAC (second column). The fibre field for anatomy 1 is expressed in the UAC basis with UAC angle ($\theta$), shown in both 2D and 3D, where $\theta$ is measured from the $\alpha _{\text{LA}}$ axis. This fibre field is then mapped using UAC to the target anatomy, to give fibre field 1 on anatomy 6 (A6, F1)

Fibres were mapped between atrial anatomies using the universal atrial coordinate system. First, UAC were calculated for source and target anatomies as described in the "Atrial Coordinates" section and the Supplementary Material, and as shown in Fig.  3. Local orthonormal bases were constructed using UAC for each element on the source and destination meshes.^33^ The fibre vector field on the source mesh was then expressed in the UAC basis using barycentric interpolation. For each element on the destination mesh, the vector direction in UAC was projected from the source geometry to the target geometry in Cartesian space.

Fibre angles ($\theta$) were expressed in the UAC basis. For the LA, angles were calculated from the $\alpha _{\text{LA}}$ axis, so that $\theta \sim 0$ (or $\pi$) for fibres in the lateral–septal direction and $\theta \sim \pi /2$ for the posterior–anterior direction. Figure  3 shows the UAC fibre angles for a source LA mesh mapped to a target LA mesh. For the RA, angles were calculated from the $\beta _\text{{RA}}$ axis, so that $\theta \sim 0$ (or $\pi$) for fibres in the IVC-SVC direction, and $\theta \sim \pi /2$ for fibres in the lateral–septal direction. Fibre vector fields are defined between 0 and $\pi$ since fibre vectors do not have a sense; as such, $\theta$ values were mapped to fall between 0 and $\pi$. We plotted probability density histograms of UAC fibre angle for the LA and RA endocardial and epicardial walls separated into the posterior wall region, the roof and the anterior wall for the LA; or septal, roof and lateral wall for the RA. We split the LA histogram into lateral-septal fibres ($\theta < \pi /4$ and $\theta > 5\pi / 6$) and posterior–anterior fibres ($\pi /4< \theta < 5\pi /6$) and expressed these as percentages. Similarly, we split the RA histogram into IVC to SVC fibres ($\theta < \pi /4$ and $\theta > 5\pi / 6$) and fibres in the lateral–septal direction (from the lateral tricuspid valve over the roof to the septal tricuspid valve, $\pi /4< \theta < 5\pi /6$). To calculate the mean of the fibre vector fields, each anatomy and associated fibre field was registered to the same anatomy (following the steps shown in Fig.  3). We then calculated the circular mean and the circular standard deviation of the fibre vector field angles in the range 0 to $\pi$, which correctly accounts for the discontinuity in angle between 0 and $\pi$ (implemented using the scipy circmean and circstd functions^43^ with settings low = 0, high = $\pi$ to respect fibre orientation but disregard fibre direction).

Following Krueger,^21^ we compared the vector fields by calculating the angle $E_i$ between two fibre vectors $F_{i,1}$ and $F_{i,2}$ as: $E_i = \arccos ( (F_{i,1} \cdot F_{i,2}) / (\Vert F_{i,1}\Vert \Vert F_{i,2}\Vert )),$ where angles greater than $\pi /2$ were corrected as $E_i = \pi - E_i$. We then calculated the proportion of angle differences below $\pi /8$, termed $Q_{\pi /8}$^21^ These differences were calculated separately for the LA posterior wall, roof, anterior wall, LAA and PV, defined as regions in UAC, for the endocardial and epicardial surfaces. Similarly, for the RA these were calculated for the lateral wall, roof, septal wall, RAA and vena cavae.

### Fibre Visualisation

To visualise fibre fields as streamline trajectories, we adapted the methodology of Saliani *et al*.^38^ to work with the UAC system and the complexity of real fibre fields. As explained in "Fibre Mapping and Calculating Mean Fibre Fields" section, fibres were expressed in the UAC basis with a UAC fibre angle, $\theta$. Streamlines were initially calculated from a random distribution of 10,000 seed points over the atrial surface, and then a subset of the calculated streamline paths were selected to generate a streamline field with regular spacing and coverage. This calculation is described in the Supplementary Material.

### Local Activation Time Simulations

Activation time maps were calculated for each of the left and right atrial endocardial and epicardial meshes separately with fibre fields from each of the anatomies, as well as with the average fibre field and with a random fibre field to simulate the isotropic case. Equivalent simulations were performed for left and right atrial bilayer models, composed of linearly coupled endocardial and epicardial surfaces with the corresponding fibre fields to those used for the single surface cases. Bilayer meshes were constructed for the LA by duplicating the endocardial surface and projecting 0.1 mm epicardially to generate an epicardial surface, and for the RA by duplicating the epicardial surface and projecting 0.1 mm endocardially. The projection distance used here is an arbitrary value that does not represent the atrial wall thickness, which is incorporated in the model by the choice of coupling coefficient value following Labarthe *et al*.^24^ This value can be modified to reflect different coupling between the endocardial and epicardial surfaces due to changes in thickness or the presence of interstitial fibrosis. Simulations were run using the carpentry simulator (available at: https://carp.medunigraz.at/carputils/index.html), with a time step of 20 $\mu$s, using the Courtemanche human atrial model^10^ with changes representing electrical remodelling during AF,^11^,^23^ together with the monodomain model for propagation. To exclude the effects of repolarisation heterogeneity, cell model properties were either set to left or right atrial properties globally, without modelling any differences in the repolarisation properties of the pulmonary veins or appendages. Longitudinal conductivity was set to 0.4 S/m and transverse conductivity to 0.1 S/m.^3^ Simulations were run for 1 s of no pacing to reach a steady state, followed by 5 beats at a cycle length of 700 ms; an activation time map was calculated for the fifth beat. To account for conduction anisotropy, activation maps for pacing from two separate locations were calculated for each anatomy. For the left atrium, pacing was from the CS or from the right superior pulmonary vein (RSPV); and for the right atrium, from the right atrial appendage (RAA) or IVC. To ensure anatomies were paced in equivalent locations, the stimuli locations were selected manually for the first anatomy and then mapped to the other anatomies using UAC.

Spatial fields of absolute LAT differences between simulations with different fibre fields were calculated and post-processed to give the median absolute LAT difference, and maximum absolute LAT difference expressed as a percentage of the total activation time. The effects of anisotropy ratio were investigated by modifying the ratio from the baseline of 4:1 to 10:1 (longitudinal conductivity 0.4S/m, transverse conductivity 0.04S/m).

### Arrhythmia Simulations

Arrhythmias were simulated for left and right atrial bilayer models for each anatomy with fibre fields from each of the anatomies, as well as with the average fibre field and with a random fibre field to simulate the isotropic case. Cell model properties and tissue conductivities were the same as above ("Local Activation Time Simulations" section), with I$_{K1}$ conductance modified to two times the baseline value^10^ to reduce the wavelength and increase the likelihood that simulated arrhythmias were sustained.^37^ To induce AF in each of the models using an equivalent set-up, initial conditions corresponding to four spiral wave reentries were used to automatically generate arrhythmias.^27^ We used initial conditions corresponding to an activation time field with two Archimedean spirals on the posterior wall and two spirals on the anterior wall, with equal UAC spacing between spirals and with opposite chirality for adjacent spirals. Identical initial conditions were used for the endocardial and epicardial surfaces. For each simulation set-up, phase singularities were calculated for 10 s of arrhythmia data to identify centres of rotational activity and re-entrant driver locations, or until termination if this occurred before 10 s. Detected phase singularities were then post-processed to calculated spatial phase singularity density maps using our previously published methodology.^32^ We calculated the Pearson correlation coefficient between each pair of 2D phase singularity maps using the MATLAB corr2 function, where 1 is total positive linear correlation, 0 is no linear correlation, and − 1 is total negative linear correlation.

## Results

We first present the left atrial endocardial and epicardial fibre maps coloured by UAC angle ("Left Atrial Fibre Maps" section), followed by the corresponding right atrial maps ("Right Atrial Fibre Maps" section). We then calculated the average LA and RA fibre fields across the anatomies by mapping each anatomy and associated fibre field to a single LA and RA anatomy from the dataset using UAC ("Average Fibre Maps" section). The effects of fibre field on local activation time maps during pacing ("Effects of Fibre Field on Local Activation Time Maps" section) and also on electrical reentrant driver location during AF ("Effects of Fibre Field on Arrhythmia Dynamics" section) were determined.

### Left Atrial Fibre Maps

**Figure 4:**
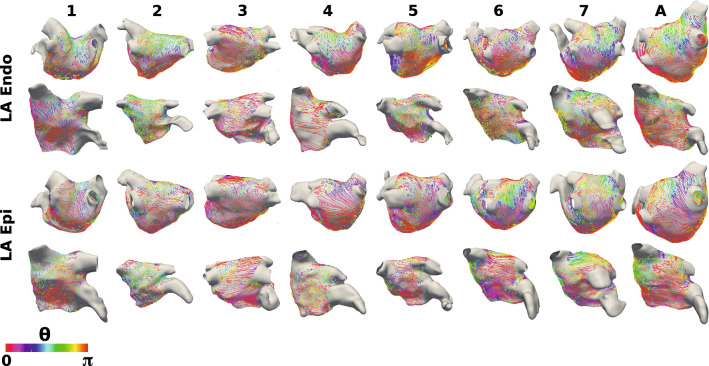
Left atrial fibre fields. Left atrial body fibre fields are displayed as streamlines coloured by UAC angle for the endocardial surfaces (top two rows) and epicardial surfaces (bottom two rows). These are shown in posteroanterior view (first and third row) and anteroposterior view (second and fourth row). Streamlines originating from the PV and LAA are omitted for visualisation purposes.

Figure 4 shows endocardial and epicardial fibre fields for seven anatomies, for which streamlines are coloured by the UAC angle, $\theta$. For fibres in the lateral-septal direction, $\theta \sim 0$ (or equivalently $\pi$, displayed as red or purple); for fibres in the posterior–anterior direction, $\theta \sim \pi /2$ (green). Visually, six of the seven anatomies (all except case 3) have posterior–anterior fibres on the roof of the endocardial surface. This is also evident from the probability density histograms in Supplementary Fig. 1 and Supplementary Sect. 1.2, and the posterior–anterior fibre percentages, which are moderate except for case 3, which has more fibre vectors in the lateral–septal direction: F1 69.0%, F2 74.6%, F3 29.3%, F4 58.2%, F5 50.2%, F6 38.8%, F7 62.8%, FA 59.7%. For the epicardial surface, the roof of anatomies 1, 2, 6 and 7 exhibit fibres in the posterior–anterior direction (posterior–anterior percentages: F1 46.4%, F2 50.5%, F6 52.9%, F7 54.8%), while for anatomies 3, 4 and 5, there is more variability in roof fibre direction and lower posterior-anterior fibre percentages (F3 29.8%, F4 39.8%, F5 39.3%, see Supplementary Fig. 2). For each of the anatomies, there are regions of the posterior wall with fibres orientated in the lateral-septal direction on both the endocardial (lateral-septal fibre percentages: F1 $73.1\%$, F2 $77.8\%$, F3 $88.2\%$, F4 $84.6\%$, F5 $66.6\%$, F6 $51.7\%$, F7 $69.0\%$, FA $94.8\%$) and epicardial surfaces (F1 $72.4\%$, F2 $70.6\%$, F3 $81.4\%$, F4 $78.2\%$, F5 $72.0\%$, F6 $72.0\%$, F7 $65.1\%$, FA $92.1\%$). The anterior wall fibres show more variability between the anatomies, with some showing predominantly posterior–anterior fibres, some lateral–septal and for some $\theta \sim \pi /4$. This is evident from the range of lateral–septal fibre percentages: F1 $70.0\%$, F2 $50.9\%$, F3 $82.8\%$, F4 $68.5\%$, F5 $73.9\%$, F6 $58.3\%$, F7 $65.2\%$, FA $76.7\%$. Endocardial and epicardial surfaces exhibit regions of similar fibres and also regions with perpendicular fibres at different locations across the anatomies.

### Right Atrial Fibre Maps

**Figure 5:**
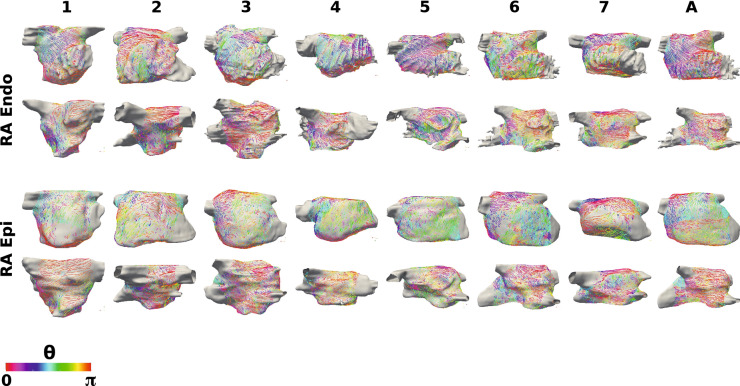
Right atrial fibre fields. Right atrial body fibre fields are displayed as streamlines coloured by UAC angle for the endocardial (top two rows) and epicardial (bottom two rows) surfaces. These are shown in lateral-septal view (first and third row) and septal-lateral view (second and fourth row). These are orientated such that the IVC-SVC universal atrial coordinate axis is aligned with the *x*-axis and the lateral-septal coordinate is aligned with the *y*-axis. UAC angles of 0 or $\pi$ correspond to the horizontal direction (aligned with the IVC-SVC coordinate axis), and UAC angles of $\pi /2$ correspond to the vertical direction (aligned with the lateral-septal coordinate axis). Streamlines originating from the SVC, IVC, RAA and CS are omitted for visualisation purposes.

Figure 5 shows the corresponding right atrial body fibre fields on the endocardial and epicardial surfaces of the seven anatomies, for which streamlines are coloured by the RA UAC angle, $\theta$. Similar to the LA visualisation, for fibres in the IVC to SVC direction $\theta \sim 0$ (or equivalently $\pi$, displayed as red or purple); for fibres in the lateral-septal direction (from the lateral tricuspid valve over the roof to the septal tricuspid valve) $\theta \sim \pi /2$ (green). Several of the anatomies (2, 4, 6 and 7) exhibit lateral-septal endocardial fibres running along the trabeculated pectinate muscles (see Supplementary Fig. 3 and Supplementary Sect. 1.4). Regions of the epicardial fibre field on the lateral wall are also in the lateral-septal direction for each of the anatomies (lateral-septal percentages: F1 42.9%, F2 50.2%, F3 40.5%, F4 49.7%, F5 71.6%, F6 71.8%, F7 35.5%, FA 55.6%; see Supplementary Fig. 4 and Supplementary Sec. 1.4). The septal wall close to the roof demonstrates SVC-IVC fibre orientation on both surfaces across the anatomies. Closer to the tricuspid valve, the septal wall shows more variability in preferential direction between the anatomies.

### Average Fibre Maps

**Figure 6:**
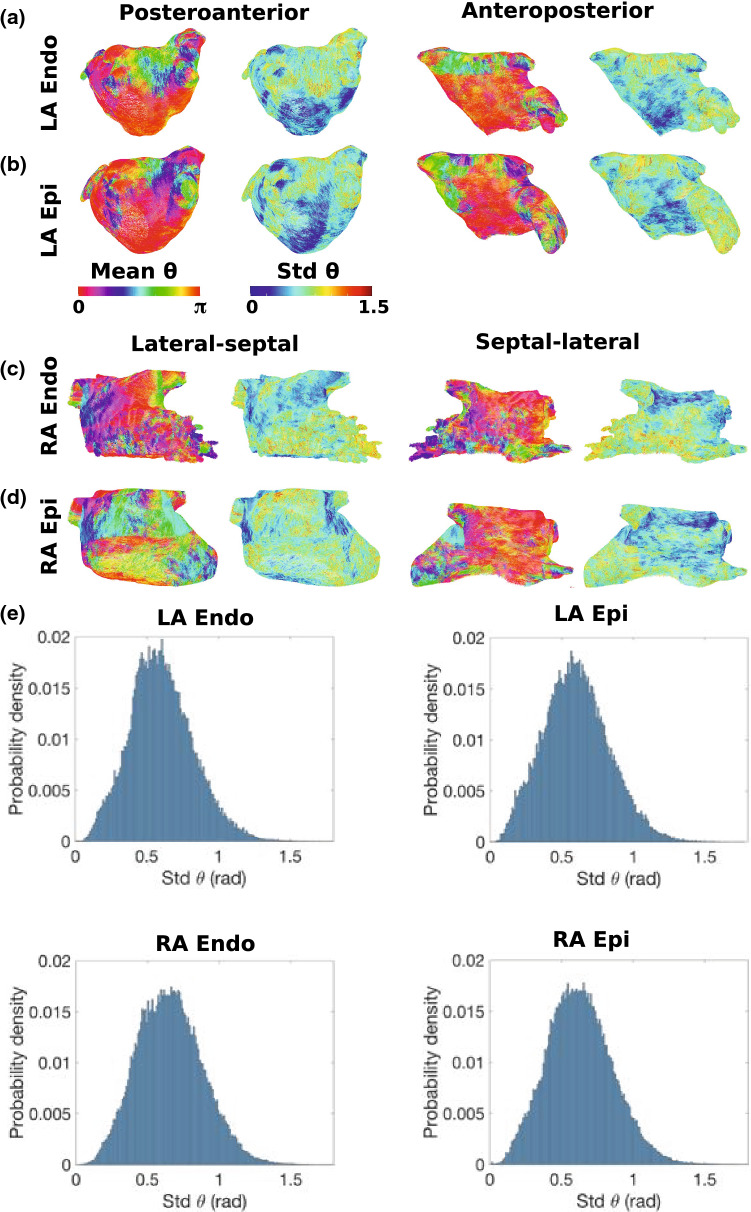
Average fibre fields. Mean and standard deviation fibre fields are shown for the following surfaces: (a) LA endocardium, (b) LA epicardium, (c) RA endocardium, (d) RA epicardium. These maps are shown in posteroanterior view for the LA (equivalently lateral-septal view for the RA) in the first and second columns, and in anteroposterior (or septal-lateral) view in the third and fourth columns. The first and third columns show the mean fibre angle field, while the second and fourth columns are the standard deviation of this field. (e) Probability density plots for standard deviation of fibre angle for each of the surfaces.

Figure 6 shows average and standard deviation fibre fields for the LA and RA endocardial and epicardial surfaces. In agreement with the visual assessment in "Left Atrial Fibre Maps" section, the average LA fibre fields demonstrate regions of posterior-anterior fibre direction on the roof (posterior-anterior fibre percentage for LA endocardium: 59.7%; LA epicardium 38.9%). For the average LA fibre field, a greater proportion of the fibres are orientated in the lateral-septal direction on the posterior wall than the individual fibre fields: endocardial posterior wall lateral-septal fibre percentages for the average field: 94.8%, compared to range for individual fibre maps: 51.7–88.2%; epicardial: 92.1%, compared to range for individual fibre maps: 65.1–81.4%. The standard deviation map shows there is greater variation in fibre direction on the roof than on the posterior wall, which agrees with the visual assessments. Probability density plots for the standard deviation of $\theta$ are given in Figure 6e. The average fibre fields for the lateral RA surfaces exhibit areas of lateral-septal fibres (dark blue–green) as well as some areas of SVC-IVC fibres in red. The lateral-septal direction is orientated along the trabeculated pectinate muscle structure; whereas, the SVC-IVC direction is parallel to the crista terminalis direction. Differences between the individual fibre fields mean the average field does not exhibit clear lateral-septal endocardial fibres running along the trabeculated pectinate muscles. As such, the average fibre field has a lower percentage of lateral–septal fibres than most of the individual fibre fields: F1 28.0%, F2 38.4%, F3 39.4%, F4 46.2%, F5 38.6%, F6 48.9%, F7 43.6% and FA 3.02% (see Supplementary Figs. 3, 4 and Supplementary Sect. 1.4). The RA septal wall demonstrates large areas of fibres in the SVC-IVC direction, particularly close to the roof where the standard deviation of the fibre field is lower.

Individual fibre fields were compared to the average fibre field and to the Labarthe *et al.* fibre field in Supplementary Section 1.5, Supplementary Tables 1, 2, Supplementary Figs. 5 and 6. In general, there is a lower agreement between the different fibre fields in the LAA and PV regions than on the LA wall (posterior, roof and anterior regions), and individual fibre fields are more similar to their average fibre field than to the Labarthe *et al*. rule-based fibre field (see Supplementary Table 1, Supplementary Fig. 5).

### Effects of Fibre Field on Local Activation Time Maps

For each of the left and right atrial bilayer models, 126 activation time maps were calculated, corresponding to the seven different anatomies, with nine different fibre fields (one from each of the seven anatomies, the average field and the isotropic case), and with two different pacing locations. To assess the individual contribution of the endocardial and epicardial fibre fields to LAT maps, the same set-up of activation maps were also calculated for left and right atrial endocardial and epicardial surface models. In total, 756 activation time maps were calculated. Example activation time for each of the anatomies and fibre fields are shown in Supplementary Figs. 7 and 8.

Each LAT field was compared to the LAT field corresponding to the same anatomy and pacing location with the original fibre field for that anatomy to determine the optimal fibre field, as well as the effects of including anisotropy.

**Figure 7:**
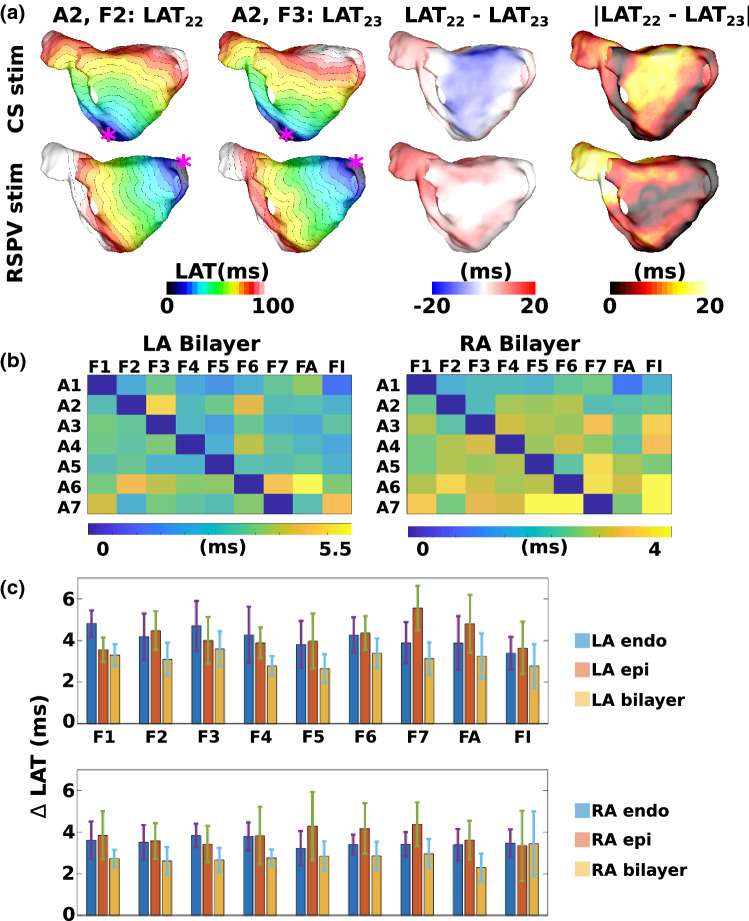
Local activation time maps depend on fibre field. (a) Example LAT maps with pacing from the CS (top) and RSPV (bottom) for either the patient-specific fibre field (first column) or a fibre field mapped from a different anatomy (second column). Spatial maps of the differences (third column) and absolute differences between these LAT maps were constructed (fourth column), and the median of these two fields was calculated. Isochrone lines are at 5ms spacing. (b) Median absolute LAT differences are shown for each anatomy (rows A1–A7) between each fibre field (columns F1–F7, FA and FI) and the gold standard LAT map (corresponding to the fibre field for that anatomy), for LA bilayer (left) and RA bilayer (right) simulations. (c) Bar charts showing the mean and standard deviation of the median LAT differences for each fibre field (F1–F7, FA and FI), calculated across the 7 anatomies. These are given for the endocardial, epicardial and bilayer simulations for the LA (top) and RA (bottom). FA corresponds to the average fibre field; FI corresponds to the isotropic case.

Figure 7a shows an example with pacing from either the CS (first row) or RSPV (second row) with the original fibre field (first column) or a fibre field from a different anatomy (second column); these are used to calculate a spatial map of LAT differences (third column). For each simulation, we calculated the median of the absolute LAT differences. For each fibre field, we calculated the mean and standard deviation of the median absolute LAT differences across simulations with that fibre field. The median of these spatial maps for the two pacing sites across the different combinations of anatomy and fibre field for the LA and RA bilayer simulations are shown in Figure 7b. For the LA bilayer simulations, fibre field 5 has the smallest average LAT difference across the seven anatomies (mean 2.67 ± 0.69, range of means for other fibre fields: 2.78–3.60 ms). For the RA bilayer simulations, the average fibre field has the smallest average LAT difference (mean 2.29 ± 0.67 ms, range of means for other fibre fields: 2.60–3.44 ms). For the isotropic case, the average LAT differences were relatively small: LA bilayer model: 2.78 ± 1.06 ms; RA bilayer model: 3.44 ± 1.55 ms.

Supplementary Figure 9 shows the equivalent plots for maximum absolute LAT differences. For the LA bilayer simulations, fibre field 5 has the smallest average maximum LAT difference of the eight fibre fields (1–7 and the average) across the seven anatomies (mean $12.7 \pm 1.68\%$, range of means for other fibre fields: 13.8–16.6%). For the RA bilayer simulations, fibre field 6 has the smallest average maximum LAT difference (mean $11.9\pm 2.96\%$, range of means for other fibre fields: 11.9–15.0%). For the isotropic case, the average maximum LAT differences were: LA bilayer model: $12.4 \pm 2.75\%$; RA bilayer model: $13.6 \pm 3.08\%$.

The equivalent measures for the endocardial and epicardial simulations are shown in the bar charts in Fig. 7c. For the LA endocardium, the isotropic case is optimal (mean 3.39 ± 0.78 ms, range of means for other fibre fields: 3.81–4.82 ms), while for the LA epicardium, fibre field 1 is optimal because it has the smallest average LAT difference (mean 3.56 ± 0.59 ms, range of means for other fibre fields: 3.65–4.80 ms). Correspondingly, for the RA endocardium, fibre field 5 is optimal (mean 3.22 ± 0.82 ms, range of means for other fibre fields: 3.39–3.84 ms); for the RA epicardium, the isotropic case is best (mean 3.34 ± 1.68 ms, range of means for other fibre fields: 3.42–4.38 ms).

LAT fields for the endocardial LA and epicardial RA surface simulations were compared to the corresponding LAT values for the LA bilayer and RA bilayer simulations, for each combination of anatomy and fibre field. For the LA, the median LAT difference between the endocardial surface LAT field and the LA bilayer LAT field was in the range: 0.79–4.62ms (mean: 2.09ms). For the RA, the median LAT difference between the epicardial surface LAT and the RA bilayer LAT field was in the range: 0.73–4.42ms (mean: 2.31ms). As such, the differences between the single surface model LAT fields and bilayer LAT fields are of a similar magnitude to the differences between the patient-specific and mapped fibre fields for the bilayer model.

The effects of anisotropy ratio on LAT maps was investigated by increasing the anisotropy ratio from 4:1 to 10:1. Supplementary Figure 10 shows that increasing the anisotropy ratio increases LAT differences between different fibre field simulations. The range of mean values for the median absolute LAT differences increased from 2.66–3.60 to 4.51–5.77 ms for the LA, and from 2.29–2.96 to 3.75–4.88 ms for the RA as conductivity was increased from 4:1 to 10.1. Similarly, the range of mean values for the maximum absolute LAT differences as a percentage of total activation time increased from 12.8–16.6 to 19.7–23.3% for the LA, and from 11.90–15.0 to 17.2–20.7% for the RA.

### Effects of Fibre Field on Arrhythmia Dynamics

**Figure 8:**
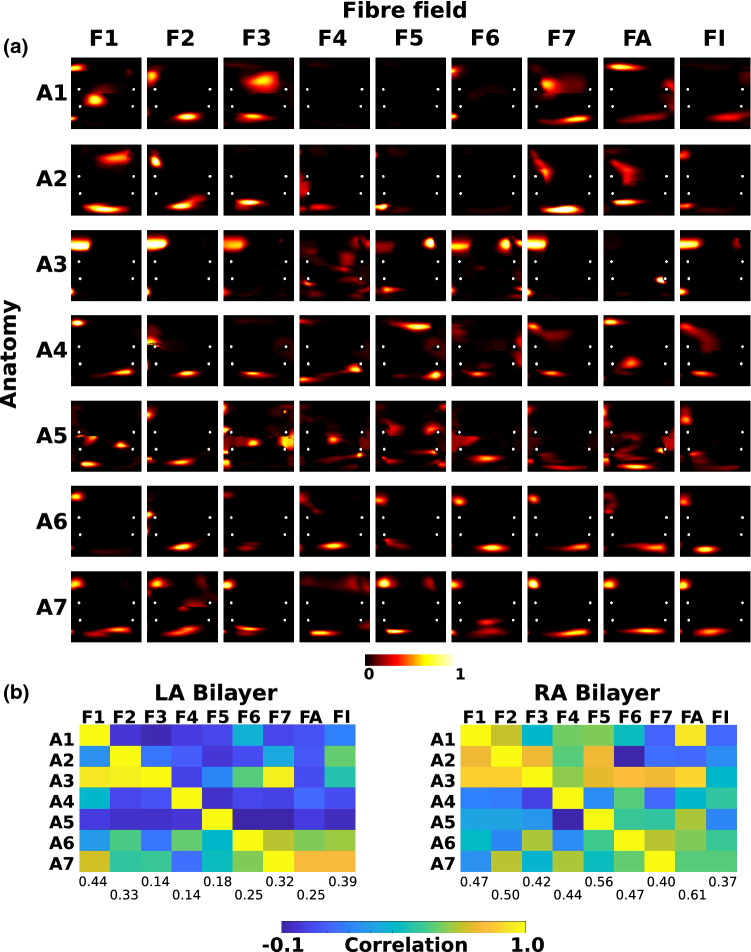
Phase singularity density maps depend on fibre field. (a) PS density maps are shown in UAC for the endocardium of LA bilayer simulations for each of the 7 anatomies (rows A1–A7) and for each fibre map (columns F1–F7, FA and FI). (b) Matrices showing correlations between phase singularity density maps for each fibre field (columns F1–F7, FA and FI) and the gold standard map (corresponding to the fibre field for that anatomy), for LA bilayer (left) and RA bilayer (right) simulations. Mean correlation values across the anatomies are shown for F1–F7, FA and FI below the matrices. FA corresponds to the average fibre field; FI corresponds to the isotropic case.

For the seven different anatomies with the nine different fibre fields (one from each of the seven anatomies, the average field and the isotropic case), AF was simulated for both the LA and RA bilayer models, resulting in 126 AF simulations, which were post-processed to identify locations of electrical drivers by calculating PS density maps. Similar to the analysis of the LAT simulations, each PS density map was compared to the corresponding map for the same anatomy with the fibre field for that anatomy, which was considered to be the gold standard AF simulation output. Each map was expressed in UAC and 2D correlation coefficients were computed between each map and the gold standard map for a given anatomy. Figure 8a shows the PS density maps for the endocardium of the LA bilayer model simulations for which PS maps in each row are compared to the corresponding map. Visually, PS density maps vary with both anatomy and with fibre map.

This is quantified by calculating correlation matrices (Pearson correlation coefficient, corr2 in MATLAB) between each pair of LA and RA bilayer fibre map simulations, shown in Fig. 8b. Correlation coefficients close to 1 indicate similar PS density maps. The mean correlation coefficients calculated across anatomies for each fibre map are also given in Fig. 8b. Fibre field 1 has the highest mean correlation coefficient for the LA bilayer simulations (0.44, range 0.14–0.39), while the average fibre field has the highest for the RA bilayer simulations (0.61, range 0.37–0.56). This shows that these fibre fields result in AF PS density maps closest to the gold standard PS density maps calculated with the correct fibre field for each anatomy. For the LA bilayer model, the isotropic case ranked second (correlation for isotropic: 0.39, fibre field 1:0.44, other fibre fields: 0.14–0.33); while for the RA bilayer case, the isotropic simulations ranked last (0.37, others: 0.40–0.61).

The average number of PS for each fibre field are similar for the LA fibre fields 1–7 (range 1.97–2.42), but higher for the average fibre field (4.88). For the RA, the average number of PS is similar across all fibre fields (range 1.95–3.09).

To investigate the effects of using rule-based approaches compared to DTMRI fibre fields on arrhythmia dynamics, AF was simulated in LA bilayer models for each of the anatomies with the Labarthe *et al*^24^,^25^ rule-based fibre atlas. These results are presented in the Supplementary material Section 1.7 & Supplementary Fig. 11.

## Discussion

### Main Findings

In this study, we developed a methodology for constructing an average fibre atlas—for use in computational modelling studies and to account for anisotropy in clinical measurements—from the high resolution DTMRI dataset of Pashakhanloo *et al*.^29^ We expressed each fibre field in a UAC system and mapped each anatomy and its associated fibre field to the same atrial anatomy on which an average fibre field was calculated. We tested the effects of fibre field on predicted activation times. The impact on average LAT difference was small with a range of mean local activation time differences for LA fields: 2.66–5.56 ms, and for RA fields: 2.29–4.38 ms, which supports the use of atlas based fibres for activation simulations. However, maximum LAT differences were larger, and differences increase further when anisotropy increases, which may be particularly important in the case of regional differences in anisotropy. We next assessed the effects of fibre field on predicted electrical driver locations by calculating phase singularity density maps for bilayer AF simulations with the different combinations of atrial anatomies and fibre fields. Fibre field had a larger effect on predicted AF phase singularity density maps than on activation times (range of PS density map correlations: LA: 0.14–0.44, RA: 0.40–0.61). Fibre field 1 had the highest mean correlation coefficient for phase singularity density maps for the LA bilayer simulations, while the average fibre field had the highest for the RA bilayer simulations. As such, in the case that a single atlas fibre field is used for simulation studies, we suggest using the fibre field corresponding to anatomy 1 for LA simulations, and the average of the fibre fields for RA simulations.

### Fibre Variability

Figures 4 and  5, together with Supplementary Figs. 1–4, show that both left and right atrial endocardial and epicardial fibre fields vary across anatomies. This results in regions of high standard deviation for the atlas maps shown in Fig. 6. Interestingly, for the LA, one of the individual patient fibre fields is optimal; whereas, for the RA, the average fibre field is optimal. This could in part be because there are large differences between patients in left atrial fibre fields, representing separate populations, and as such an average fibre field across all cases is not optimal. Alternatively, this may suggest that the large differences in LA fibre fields mean that patient-specific LA fibre fields are required for arrhythmia prediction; whereas, for the RA, a rule-based or atlas approach to fibre inclusion is more suitable, and the inclusion of anisotropy is important. In addition, different arrangements of fibres at the junction of the left atrial body and pulmonary veins have been reported in the literature^16^,^18^,^42^ and these differences have been shown to affect arrhythmia dynamics.^31^.

### Comparison to Other Methodologies and Atlases for Personalising Atrial Fibre Fields

Methodologies for personalising atrial fibre fields fall broadly into two categories: rule-based methodologies and registration-based methodologies. Rule-based methodologies generate a new fibre field for each anatomy following a sequence of rules, typically based on histological descriptions. Alternatively, registration techniques map fibres to an anatomy from a reference atlas, which may itself be constructed using a rule-based approach. Our methodology falls somewhere between the two categories: we first construct a fibre atlas from DTMRI data and then *register* this atlas using UAC to a new anatomy; equally, expressing the DTMRI atlas in UAC creates a *rule-based* approach for assigning fibres to a new model in a UAC basis.

Fastl *et al*. developed a rule-based approach for assigning endocardial and epicardial fibre fields to patient-specific left atrial anatomies based on local solutions of Laplace’s equation to generate a field that matches morphological descriptions.^12^,^16^,^17^ The methodology of Fastl *et al*. is similar to the current methodology because both use a sequence of Laplace solves; however, Fastl *et al*. divide the LA into a much larger number of regions (151) using a sequence of 122 landmark points with 272 auxiliary lines, chosen to match descriptions of atrial histology from the literature. Labarthe *et al*. also developed a semi-automatic rule-based technique for assigning fibre fields that broadly match histological descriptions by splitting the anatomy into cylindrical structures, defining cylindrical fibres on these structures or filling the fibre field using an inpainting technique.^24^,^25^ Our current study assigns fibres to both the left and right atria, uses fewer landmark points (3) and incorporates fibre fields from DTMRI data.^29^.

Roney *et al*. registered the bilayer fibre atlas of Labarthe *et al*. to patient-specific bilayer models using the UAC system.^24^,^33^ Our current methodology significantly extends this study by fixing the locations of key anatomical structures, including the PV, vena cava, appendages and CS, to their average location across the dataset to generate an atlas coordinate system, and uses high-resolution DTMRI fibres. McDowell *et al*. use an image-based registration method to transform the atlas geometry and fibre field of Krueger *et al*. to target geometries.^20^,^28^ Their technique involves assigning a large number of landmarks manually before applying a 3D thin plate spline transformation and a large deformation diffeomorphic metric mapping.^4^.

We compared the average fibre atlas constructed in this study to the fibre atlas of Labarthe *et al*.^24^,^25^ Supplementary Table 1 and Supplementary Fig. 5 show that there is generally a lower agreement between the different fibre fields in the LAA and PV regions than on the LA wall, and that individual fibre fields are more similar to their average fibre field than to the Labarthe *et al*. rule-based fibre field. This atlas was constructed based on histological descriptions of atrial fibre structures. Electrical propagation using the Labarthe *et al*. fibre atlas is smoother than propagation using any of the fibre fields in the current study (compare the isopotential plots in Supplementary Fig. 11A). This is to be expected as rule-based fibre fields typically include smooth changes in fibres,^13^,^20^ while DTMRI data exhibits more local variation due to physiological heterogeneity, and measurement noise.

### Implications for Patient-Specific Modelling Pipelines

Patient-specific models may be used to predict simple paced activation patterns^9^ or arrhythmia dynamics and ablation outcome.^6^ Figure 7 shows that paced activation maps at a cycle length of 700 ms vary with atrial fibre field; however, these differences are relatively small (median LAT difference < 5.5$\%$ of total activation time). This suggests that the range of observed human atrial fibre fields does not have a large effect on global activation pattern for pacing rates close to sinus rhythm. We also considered the isotropic case for each anatomy to investigate the effects of fibre inclusion on activation patterns, demonstrating that mean LAT differences are small, but accurate fibres are required to capture local activation patterns and local heterogeneity. As expected, paced activation times are robust on average to physiological variation in fibre orientation and error introduced by rule-based methods is unlikely to have a significant impact on the activation pattern indicated by small median absolute differences. The maxima of the absolute LAT difference maps are larger, suggesting that fibre fields are more important for assessing extreme results. The differences between the single surface model LAT fields and bilayer LAT fields for a given fibre field are of a similar magnitude to the differences between the patient-specific and mapped fibre fields for the bilayer model, suggesting that bilayer models are required to capture the combined effects of endocardial and epicardial fibres. As such, we used bilayer models—and not single surface models—for the arrhythmia simulations.

Differences in simulation output between different input fibre fields for arrhythmia simulations and predicted electrical drivers are more pronounced. Figure 8 demonstrates that the predicted locations of electrical drivers depend on both anatomy and fibre field (range of mean correlation coefficients for different fibre fields as anatomy varies: 0.14–0.44; for different anatomies as fibre field varies: − 0.05 to 0.54). Modifying the input fibre field choice changes both the number of drivers and also their location (see Fig. 8). This highlights the importance of appropriately incorporating fibre fields in atrial simulations for predicting arrhythmia mechanisms and ablation outcomes. We also considered the isotropic case for each anatomy, where we found that the inclusion of anisotropy was more important for determining the RA than the LA arrhythmia pattern. For the LA bilayer model, the isotropic case ranked second; while for the RA bilayer case, the isotropic simulations ranked last, demonstrating the importance of fibre inclusion for RA simulations. Since fibre direction had a greater effect on arrhythmia prediction than on activation map prediction for slow pacing rates, we suggest using the optimal fibre fields for the bilayer arrhythmia prediction experiments; fibre field 1 for LA simulations, and the average fibre field for RA simulations.

Patient-specific structural fibre information is not available clinically due to limitations in imaging modalities. An alternative strategy for personalising atrial fibres is to measure patient-specific electrical anisotropy;^35^ however, this is also challenging and time consuming to incorporate in standard clinical procedures as it requires pacing from three different locations to fix the longitudinal speed, transverse speed and conduction direction. In addition, the requirement of invasive measurements also precludes this approach from pre-procedural clinical modelling studies. A fibre atlas constructed from either structural data, as in the current study, or electrical data could be used for interpreting electrical measurements. For example, conduction velocity measurements exhibit directional dependency, which could be accounted for using a fibre atlas to estimate the underlying longitudinal and transverse components of conduction velocity,^35^ which is particularly important when correlating conduction properties with underlying tissue properties; for example, fibrosis distribution.^14^.

Constructing atrial models at scale requires an automated technique for incorporating fibre direction. Our method requires the manual selection of one point for the LA and two points for the RA and is otherwise fully automated. Expressing the atrial fibre fields and the average field in the UAC basis makes it possible to register this fibre field to a target anatomy that is also expressed in UAC. Due to the sensitivity of predicted driver locations to fibre field, and because patient-specific fibre fields are generally not available, we recommend running AF simulations with different input fibre fields to gauge the range of expected behaviours, subject to computational tractability. The dependence of AF driver location on atrial fibre field is likely to decrease with the addition to models of fibrotic remodelling and electrical heterogeneity as properties of these features may anchor re-entry.^40^ future studies should investigate the strength of these relative dependencies.

### Limitations

The main limitation of this study is the small sample size (seven anatomies). Increasing the number of anatomies included in the average LA fibre field calculation could result in a fibre field that is closer to the others in the population than any individual fibre field. We only considered seven of the eight biatrial anatomies from the study of Pashakhanloo *et al*.^29^ because for one of the atria we were unable to achieve a segmentation that could be used for UAC calculation for the endocardial wall. One limitation of the UAC system is that there is a discontinuity along the lateral and septal boundaries for the LA (and equivalently along the boundary paths from the SVC/IVC to the tricuspid valve for the RA). Fibres along these boundaries are not well defined in the UAC system; in the case that UAC were used for mapping, these boundary fibres were replaced by their closest non-boundary neighbour. In addition, since streamlines were calculated in 2D UAC and then mapped to 3D, their trajectories were discontinuous across these boundaries. Our current implementation of UAC together with the fibre fields in this study assumes the presence of four PV, and is not currently able to work with 3 or 5 vein atria, which make up approximately $30\%$ of cases.^30^ We only considered monoatrial models with homogeneous conductivity values; however, the inclusion of interatrial connections and specialised atrial structures may increase LAT differences and increase the need for personalised fibres. Interatrial connections could be included between the LA and RA bilayer meshes using a rule-based approach.^24^,^44^ To create the endocardial and epicardial patient-specific fibre maps, we had to differentiate between the two surfaces. The average wall thickness for the Pashakhanloo *et al*. dataset is 2.73mm (5–95% quartiles: 0.98–4.38 mm), which is 4.34 voxels (5–95% quartiles: 1.56–6.96 voxels).^29^ As such, the resolution of the DTMRI is sufficient for differentiating the two fibre fields across the majority of the atrium.^45^ Instead of including the same fibre atlas in all patient-specific models, future work could investigate choosing the fibre map corresponding to the atrial anatomy closest in morphology to the target atrial anatomy, where atrial morphologies could be compared using a principal component analysis.^41^ The current UAC system could be extended to include a transmural coordinate by performing a Laplace solve with zero on the endocardial surface and one on the epicardial surface, following Bishop *et al*.,^5^ to calculate an average volumetric fibre map. Finally, to assess the effects of variability and uncertainty of fibre field on modelling predictions, fibre angles could be post-processed by extending our previous principal component analysis^8^ to work for a circular variable.

### Conclusions

We have developed a methodology for registering atrial fibre fields to construct an average fibre atlas from the high resolution DTMRI dataset of Pashakhanloo *et al*.^29^ We have made this atlas, together with the associated atrial coordinates required for registration, available online. The impact of fibre field on average paced activation times was small, supporting the use of atlas based fibres for activation simulations. Arrhythmia dynamics were more dependent on fibre field, suggesting that atrial fibre fields should be carefully assigned to patient-specific arrhythmia models. For LA bilayer model AF simulations, fibre field 1 was optimal; whereas for RA bilayer models, the average fibre field was optimal. As such, in the case that a single atlas fibre field is used for simulation studies, we suggest using the fibre field corresponding to patient 1 for LA simulations, and the average of the fibre fields for RA simulations.

## Electronic supplementary material

Below is the link to the electronic supplementary material.

Electronic supplementary material 1 (PDF 9116 kb)
