# Supplementary material for: Constructing a Human Atrial Fibre Atlas
Source: Ann Biomed Eng. 2020 May 26;49(1):233–50. doi: 10.1007/s10439-020-02525-w (PMC7773625; doi:10.1007/s10439-020-02525-w)
Supplement: Supplementary file 1 — Electronic supplementary material 1 (PDF 9116 kb) [file 10439_2020_2525_MOESM1_ESM.pdf]

Constructing a Human Atrial Fibre Atlas

Caroline H Roney[1], Rokas Bendikas[1], Farhad Pashakhanloo[2], Cesare Corrado[1], Edward J Vigmond[3], Elliot R McVeigh[4], Natalia A Trayanova[5], Steven A Niederer[1]

affiliations: [1]School of Biomedical Engineering and Imaging Sciences, King's College London, United Kingdom. [2] Department of Medicine, Cardiovascular Division, Beth Israel Deaconess Medical Center and Harvard Medical School, USA [3] LIRYC Electrophysiology and Heart Modeling Institute, Bordeaux Fondation, avenue du Haut-Leveque, Pessac 33600, France. [4] Department of Bioengineering, UC San Diego School of Engineering,USA. [5] Department of Biomedical Engineering, Johns Hopkins University, USA.

correspondence: Caroline H. Roney, School of Biomedical Engineering and Imaging Sciences, King's College London, United Kingdom
e-mail: caroline.roney@kcl.ac.uk

1 Supplementary Material

1.1 Methods: UAC calculation

1.1.1 Landmark selection

The original UAC construction requires the user to select three landmarks for the LA and two landmarks for the RA, and is described fully in Roney et al.[4]. To reduce variability in

UAC assignment between users, the roof landmarks for the left atrial UAC were removed by instead using properties of the left atrial anatomy. Specifically, the closest point on each of the junctions of the left (or right) superior veins with the left atrial body to the left (or right) inferior veins was calculated. The points on each of the junctions of the superior veins with the left atrial body furthest from these calculated points were then automatically selected as the roof landmarks.

1.1.2 Mapping anatomical structures to atlas coordinate locations

To create a coordinate system with anatomical structures (the PV, SVC, IVC, LAA, RAA, CS) located at the same UAC, the original UAC system was extended to include additional boundary conditions. This was implemented by first calculating the original UAC system, as described in[4], and then performing a further set of Laplace solves to modify this field.

Laplace solution values were specified along boundary paths calculated between reproducible points on anatomical structures. These points were automatically selected around each of the structures (PV, LAA, SVC, IVC, CS, RAA) using properties of the original UAC field. Specifically, these points correspond to the maxima or minima location of one of the UAC coordinates along the boundaries of the structures with the atrial body; this is shown in Figure 2 (A) of the main manuscript. These 2D landmarks were then mapped to 3D for the calculation of boundary paths. Specifically, paths between two atrial structures within the UAC system were calculated as geodesic paths on the 3D surface mesh, while paths between an atrial structure and the boundary of the UAC system (i.e. corresponding to a value of 0 or 1 for α or β) were calculated as isolines from the original UAC Laplace solves. This methodology was used because geodesic paths between atrial structures represent paths between two fixed points on the mesh; whereas, isolines constructed from the original UAC connect the atrial structures to the mesh boundaries. These geodesic paths and isolines are shown for the LA in Figure 2 (B) of the main manuscript.

For the LA, paths were calculated between locations as follows:

- Point at minimum α_{LA} on each PV and LAA to MV boundary,
- Point at maximum α_{LA} on each PV and LAA to MV boundary,
- Point at minimum α_{LA} on LSPV to point at minimum α_{LA} on LAA,
- Point at minimum β_{LA} on each PV and LAA to MV boundary,
- Point at maximum β_{LA} on LIPV, RIPV and LAA to MV boundary,
- Point at maximum β_{LA} on RSPV to point at maximumβ_{LA} on LSPV,
- Point at maximum β_{LA} on RIPV to point at maximum β_{LA} on LIPV,
- Point at minimum β_{LA} on RIPV to point at minimum β_{LA} on LIPV.

To construct an atlas UAC system, the values assigned along the boundary paths were chosen to fix the locations of the atrial structures (the PV, SVC, IVC, LAA, RAA, CS) to their average location calculated across the anatomies. The boundary paths - shown in 3D in Figure 2 (B) and in 2D in Figure 2 (C) of the main manuscript - were mapped to either horizontal or vertical lines by assigning an isovalue for one of the Laplace solves. The results of fixing these paths are indicated by the straight lines in the modified UAC system, shown in the right column of Figure 2 (C) of the main manuscript.

1.1.3 Atrial coordinate calculation

The coordinates were calculated separately for meshes corresponding to the upper and lower half plane of the coordinate system. This was necessary because boundary values along paths were not consistent between the upper and lower half planes of the coordinate system, and so discontinuities exist along the lateral and septal walls of the left atrium (and SVC/IVC to tricuspid valve paths for the right atrium). These two half planes correspond to the posterior or anterior component of the left atrial mesh, and the lateral or septal component of the right atrial mesh, and so each of the left and right atrial meshes were split into these two regions for the Laplace solves. This is shown for the posterior and

anterior LA mesh Laplace solves in Figure 2 (D) of the main manuscript. Finally, the solutions for these two meshes were combined to give the solution for the entire surface; shown in Figure 2 (E). The final 2D UAC coordinates are shown for the LA in Figure 2 (F). Equivalent steps were used for the RA, resulting in the final RA UAC given in Figure 2(G) of the main manuscript.

1.2 Methods: Fibre visualisation

Streamlines were initially calculated from a random distribution of 10,000 seed points over the atrial surface, as follows. To calculate a streamline from a given seed, the seed point was first converted from Cartesian to UAC. The triangular element enclosing the seed point was identified using the nearest element barycentre and the fibre direction for that element was used to translate the seed point location. Specifically, the seed point was translated along both the positive and negative fibre direction by an increment equal to the average UAC mesh edge length. The elements enclosing each of these two new points were determined. The UAC fibre angles of these elements were compared to the UAC fibre angle of the seed point element. If the difference in angle was less than a given threshold, the point was added to the streamline and the construction continued; whereas, if the difference was greater than the threshold, the streamline was stopped at this point. Since fibre angles are defined in the upper half plane, angular differences of greater than $\pi/2$ were converted to the absolute value of the angular difference minus π, and correspondingly, angular differences of less than $-\pi/2$ were converted to the absolute value of the angular difference plus π.

This process was repeated iteratively by translating streamline end points along the fibre direction orientated away from the current streamline trajectory, until the angle stopping criterion was reached. In each case, the UAC angle of the translated point was compared to that of the previous point, and we used an angle of 0.7 radians for the angle stopping criterion. To correctly select the fibre direction that was orientated away from the current streamline trajectory, it was necessary to include an additional criterion since

UAC angle between adjacent elements may flip from close to 0 to close to π, indicating a change in vector sense. Finally, each path of points was converted from UAC to Cartesian coordinates to determine the streamline paths on the 3D surface mesh.

To generate a streamline field with regular spacing and coverage, a subset of the calculated streamline paths was selected subject to a distance criterion. Specifically, a set of paths was constructed, starting with the longest streamline and adding to the set - subject to a distance criterion - in order of decreasing streamline length. The minimum distance between each 3D point in the streamline under investigation and the current set of streamline points was calculated. In the instance that any portions of the streamline path were within a distance threshold of the current set of streamline points, these portions of the path were removed before adding the streamline to the set of paths. This was repeated by assessing all streamlines in order of decreasing length to build up a final set with a minimum distance between all paths in the set. An example is shown in Figure 3 of the main manuscript.

1.3 Results: Left atrial fibre maps

Supplementary Figure 1 shows probability density histograms of UAC fibre angle for the LA endocardial wall separated into the posterior wall region, the roof and the anterior wall. We split the histogram into lateral-septal fibres ($\theta < \pi/4$ and $\theta > 5\pi/6$) and posterior-anterior fibres ($\pi/4 < \theta < 5\pi/6$) and expressed these as percentages. All of the roof probability density histograms exhibit UAC fibre angles in the posterior-anterior direction, except case 3 which has more fibre vectors in the lateral-septal direction: posterior-anterior fibre percentages F1 69.0$\%$, F2 74.6$\%$, F3 29.3$\%$, F4 58.2$\%$, F5 50.2$\%$, F6 38.8$\%$, F7 62.8$\%$, FA 59.7$\%$. For each of the anatomies, there are regions of the posterior wall with fibres orientated in the lateral-septal direction (lateral-septal fibre percentages: F1 73.1$\%$, F2 77.8$\%$, F3 88.2$\%$, F4 84.6$\%$, F5 66.6$\%$, F6 51.7$\%$, F7 69.0$\%$, FA 94.8$\%$). For the average fibre field, a greater proportion of the fibres are orientated in the lateral-septal direction on both the posterior and anterior walls than the individual fibre fields: poste-

rior wall FA lateral-septal fibre percentage 94.8$\%$, compared to range for individual fibre maps: 51.7-88.2$\%$; anterior wall FA 93.9$\%$, compared to range for individual fibre maps: 46.8-84.7$\%$.

For the epicardial surface probability density histograms, shown in Supplementary Figure 2, the roof of anatomies 1, 2, 6 and 7 exhibit fibres in the posterior-anterior direction (posterior-anterior percentages: F1 46.4$\%$, F2 50.5$\%$, F6 52.9$\%$, F7 54.8$\%$), while for anatomies 3, 4 and 5, there is more variability in roof fibre direction and lower posterior-anterior fibre percentages (F3 29.8$\%$, F4 39.8$\%$, F5 39.3$\%$). The anterior wall fibres shows variability between the anatomies, with some showing predominantly posterior-anterior fibres, some lateral-septal and for some $\theta \sim \pi/4$. This is evident from the range of lateral-septal fibre percentages: F1 70.0$\%$, F2 50.9$\%$, F3 82.8$\%$, F4 68.5$\%$, F5 73.9$\%$, F6 58.3$\%$, F7 65.2$\%$, FA 76.7$\%$. As for the endocardial surface, the LA epicardial fibre fields also have a greater proportion of the average fibres orientated in the lateral-septal direction on the posterior wall than the individual fibre fields (posterior wall lateral-septal fibre percentages: F1 72.4$\%$, F2 70.6$\%$, F3 81.4$\%$, F4 78.2$\%$, F5 72.0$\%$, F6 72.0$\%$, F7 65.1$\%$, FA 92.1$\%$).

1.4 Results: Right atrial fibre maps

Probability density histograms of UAC fibre angle for the RA endocardial wall are shown in Supplementary Figure 3 and for the RA epicardial wall in Supplementary Figure 4. Similar to the LA , we split the histogram into IVC to SVC fibres ($\theta < \pi/4$ and $\theta > 5\pi/6$) and fibres in the lateral-septal direction (from the lateral tricuspid valve over the roof to the septal tricuspid valve, $\pi/4 < \theta < 5\pi/6$) and expressed these as percentages. Several of the anatomies (2, 4, 6 and 7) visually exhibit lateral-septal endocardial fibres running along the trabeculated pectinate muscles; however, differences between the fibre fields mean these are not evident in the average fibre map. As such, the average fibre field has a lower percentage of lateral-septal fibres than most of the individual fibre fields: F1 28.0$\%$, F2 38.4$\%$, F3 39.4$\%$, F4 46.2$\%$, F5 38.6$\%$, F6 48.9$\%$, F7 43.6$\%$ and FA 30.2$\%$. Regions

of the epicardial fibre field on the lateral wall are also in the lateral-septal direction for each of the anatomies (lateral-septal percentages: F1 42.9$\%$, F2 50.2$\%$, F3 40.5$\%$, F4 49.7$\%$, F5 71.6$\%$, F6 71.8$\%$, F7 35.5$\%$, FA 55.6$\%$). The septal wall close to the roof demonstrates SVC-IVC fibre orientation on both surfaces across the anatomies. Closer to the tricuspid valve, the septal wall shows more variability in preferential direction between the anatomies. Average SVC-IVC for the endocardial wall are: F1 57.0$\%$, F2 60.5$\%$, F3 76.7$\%$, F4 70.9$\%$, F5 41.2$\%$, F6 59.5$\%$, F7 72.3$\%$, FA 76.7$\%$; and for the epicardial wall are: F1 74.7$\%$, F2 72.5$\%$, F3 74.3$\%$, F4 70.2$\%$, F5 43.4$\%$, F6 59.0$\%$, F7 65.0$\%$, FA 82.9$\%$.

1.5 Results: Comparing fibre fields to the DTMRI atlas and rule-based atlas

Following Krueger[1], we compared the vector fields by calculating the angle E_i between two fibre vectors $F_{i,1}$ and $F_{i,2}$ as: $E_i = \arccos((F_{i,1} \cdot F_{i,2})/(\|F_{i,1}\|\|F_{i,2}\|))$, where angles greater than $\pi/2$ were corrected as $E_i = \pi - E_i$. We then calculated the proportion of angle errors below $\pi/8$, termed $Q_{\pi/8}$[1]. These differences were calculated separately for the LA posterior wall, roof, anterior wall, LAA and PV, defined as regions in UAC, for the endocardial and epicardial surfaces. Similarly, for the RA these were calculated for the lateral wall, roof, septal wall, RAA and vena cavae.

Table 1 gives $Q_{\pi/8}$ percentages for LA endocardial and epicardial atrial regions calculated between the individual fibre fields 1-7 and the average fibre field (labelled: A1-A7) or the Labarthe et al. rule-based fibre field (labelled: L1-L7), as well as between the average fibre field and the Labarthe et al. rule-based fibre field (labelled: AL). In general, there is a lower agreement between the different fibre fields in the LAA and PV regions than on the LA wall (posterior, roof and anterior regions), indicated by the difference in $Q_{\pi/8}$ values. Individual fibre fields are more similar (higher $Q_{\pi/8}$) to their average fibre field than to the Labarthe et al. rule-based fibre field. Similarly, Table 2 gives $Q_{\pi/8}$ percentages for RA endocardial and epicardial atrial regions calculated between the individual fibre fields 1-7

and the average fibre field (labelled: A1-A7).

Supplementary Figure 5 shows box plots of angle E_i between fibre vectors for different LA endocardial maps in (A) and LA epicardial maps in (B). These plots show that the individual fibre maps are more similar to the average fibre map than to the Labarthe et al rule based map. Supplementary Figure 5 (C) and (D) show the differences between each pair of maps. Supplementary Figure 6 shows the equivalent graphs for the RA.

1.6 Results: Effects of fibre field on local activation time maps

Supplementary Figure 7 shows activation time maps for the LA anatomies and fibre maps in Figure 4 of the main manuscript. Supplementary Figure 8 shows activation time maps for the RA anatomies and fibre maps in Figure 5 of the main manuscript.

Supplementary Figure 9 shows maximum absolute LAT differences as a percentage of total activation time, equivalent to Figure 7 of the main manuscript, which shows median absolute LAT differences. For the LA bilayer simulations, fibre field 5 has the smallest average maximum LAT difference of the eight fibre fields (1-7 and the average) across the seven anatomies (mean 12.7 $\pm$ 1.68$\%$, range of means for other fibre fields: 13.8 - 16.6$\%$). For the RA bilayer simulations, fibre field 6 has the smallest average maximum LAT difference (mean 11.9$\pm$2.96$\%$, range of means for other fibre fields: 11.9 - 15.0$\%$). For the isotropic case, the average maximum LAT differences were: LA bilayer model: 12.4 $\pm$2.75$\%$; RA bilayer model: 13.6 $\pm$ 3.08$\%$.

The effects of anisotropy ratio on LAT maps was investigated by increasing the anisotropy ratio from 4:1 to 10:1. Supplementary Figure 10 shows that increasing the anisotropy ratio increases LAT differences between different fibre field simulations. The range of mean values for the median absolute LAT differences increased from 2.66-3.60ms to 4.51-5.77ms for the LA, and from 2.29-2.96ms to 3.75-4.88ms for the RA as the anisotropy ratio was increased from 4:1 to 10.1. Similarly, the range of mean values for the maximum absolute LAT differences as a percentage of total activation time increased from 12.8-16.6$\%$ to 19.7-23.3$\%$ for the LA, and from 11.90-15.0$\%$ to 17.2-20.7$\%$ for the RA.

1.7 Results: Comparing DTMRI and rule-based atlases AF properties

To investigate the effects of using rule-based approaches compared to DTMRI fibre fields, AF was simulated in LA bilayer models for each of the anatomies with the Labarthe et al[3,2] rule-based fibre atlas. Electrical propagation using the Labarthe et al fibre atlas is visually smoother than propagation using any of the fibre fields in the current study. This is evident in Supplementary Figure 11 (A), which shows isopotential maps for the patient-specific fibre field (in this case, corresponding to anatomy 3), the average LA fibre field, fibre field 1 and the Labarthe et al[3,2] rule-based fibre atlas. Phase singularity density maps calculated using the Labarthe et al[2] fibre atlas are shown in Supplementary Figure 11 (B) compared to those for the patient-specific fibre fields. The mean correlation coefficient for LA bilayer simulations for the Labarthe et al atlas is higher than for the average DTMRI LA fibre field (0.39 compared to 0.25), but lower than for the optimal DTMRI fibre field (F1: 0.44).

References

1. Krüger, M. W. Personalized multi-scale modeling of the atria: heterogeneities, fiber architecture, hemodialysis and ablation therapy 19, 2013.

2. Labarthe, S., J. Bayer, Y. Coudière, J. Henry, H. Cochet, P. Jaïs, and E. Vigmond. A bilayer model of human atria: mathematical background, construction, and assessment. Europace : European pacing, arrhythmias, and cardiac electrophysiology : journal of the working groups on cardiac pacing, arrhythmias, and cardiac cellular electrophysiology of the European Society of Cardiology 16 Suppl 4:iv21–iv29, 2014.

3. Labarthe, S., Y. Coudiere, J. Henry, and H. Cochet. A Semi-Automatic Method To Construct Atrial Fibre Structures : a Tool for Atrial Simulations. CINC - Computing in Cardiology pp. 881–884, 2012.

4. Roney, C. H., A. Pashaei, M. Meo, R. Dubois, P. M. Boyle, N. A. Trayanova, H. Cochet, S. A. Niederer, and E. J. Vigmond. Universal atrial coordinates applied to visualisation, registration and construction of patient specific meshes. Medical image analysis 55:65–75, 2019.

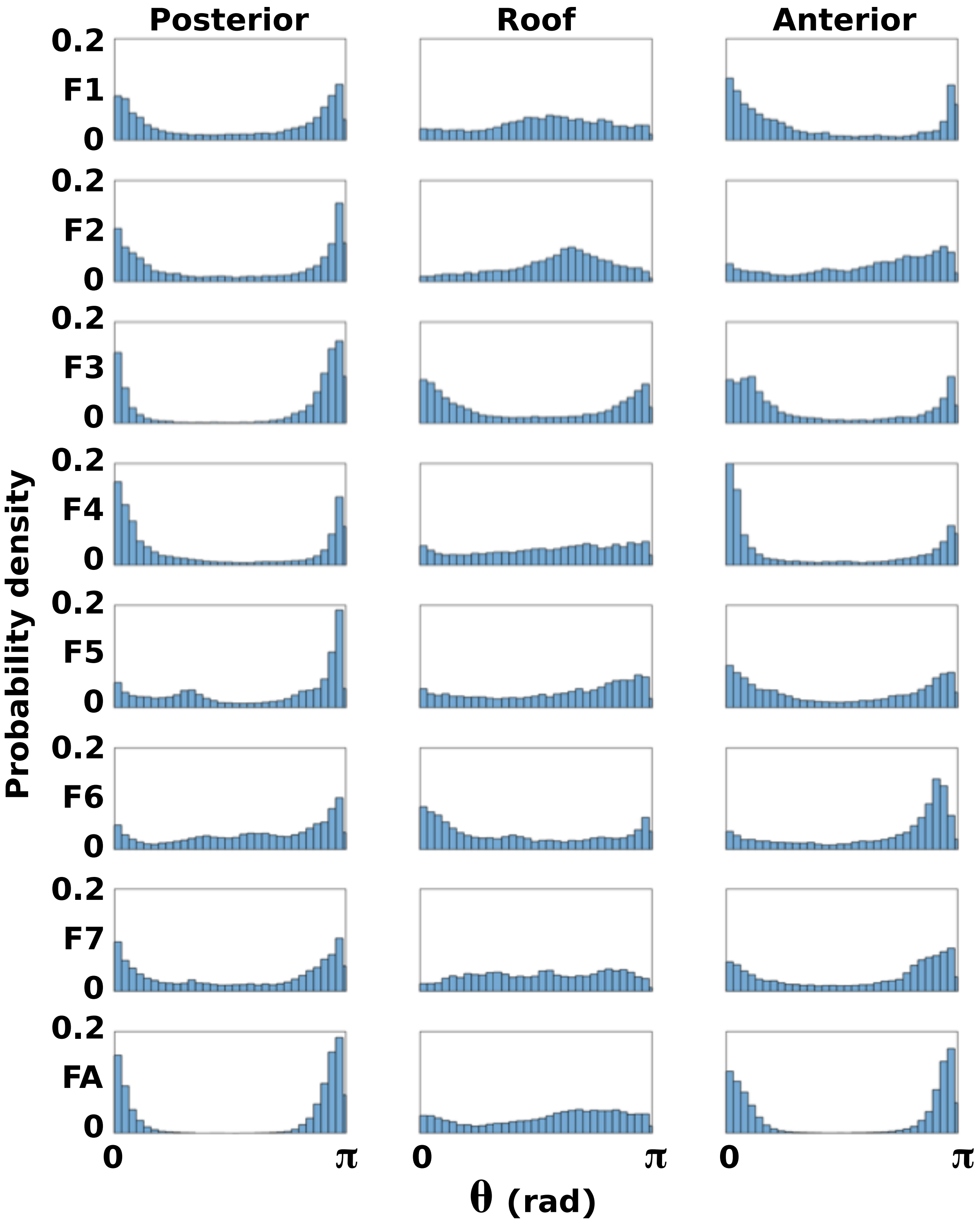

Figure 1: **UAC fibre angle distributions for the LA endocardium by atrial region.** Probability density histograms of UAC fibre angle for each LA endocardial field for the posterior wall, roof and anterior wall. Rows are for each fibre field 1-7 and the average field.

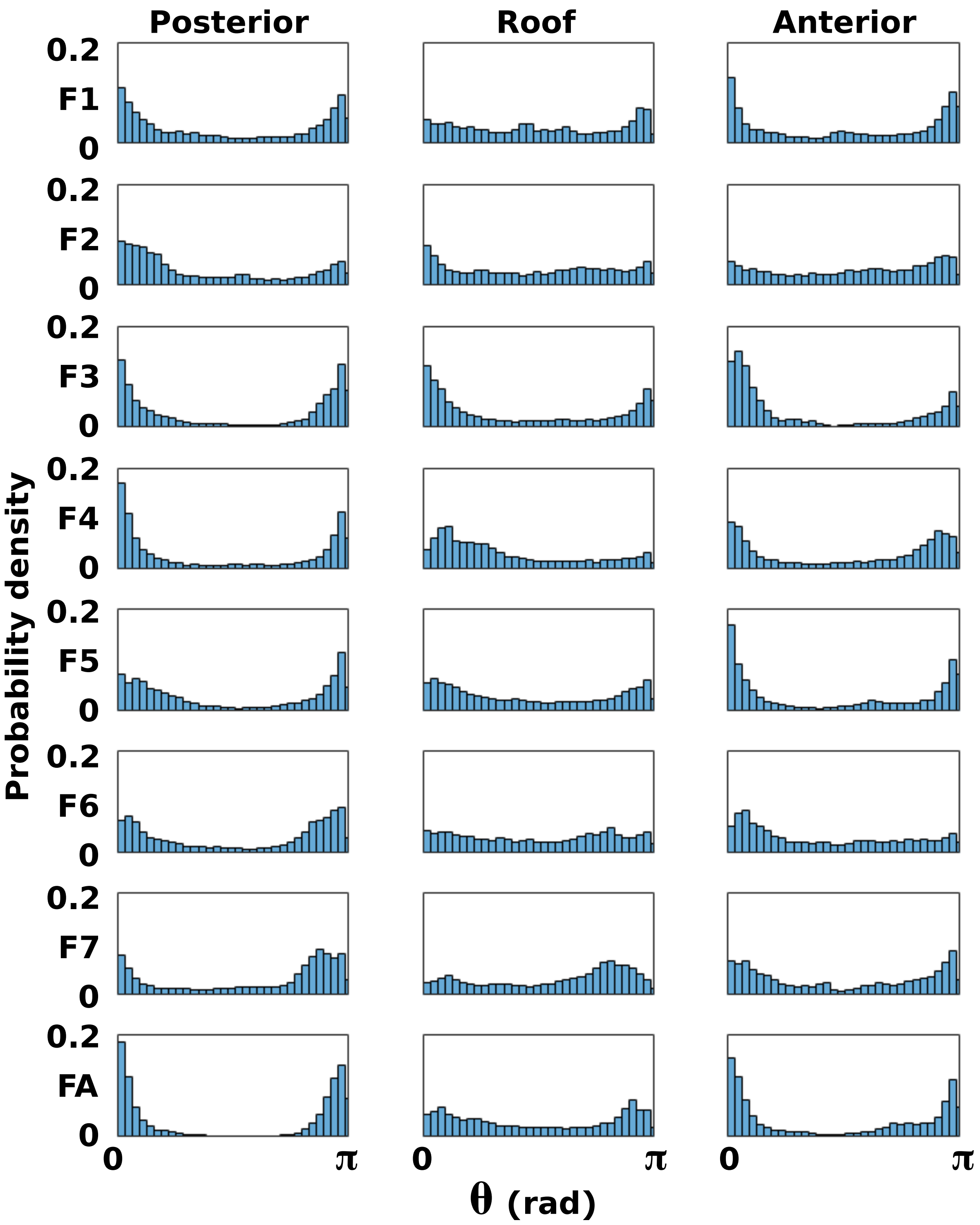

Figure 2: **UAC fibre angle distributions for the LA epicardium by atrial region.** Probability density histograms for each LA epicardial field for the posterior wall, roof and anterior wall. Rows are for each fibre field 1-7 and the average field.

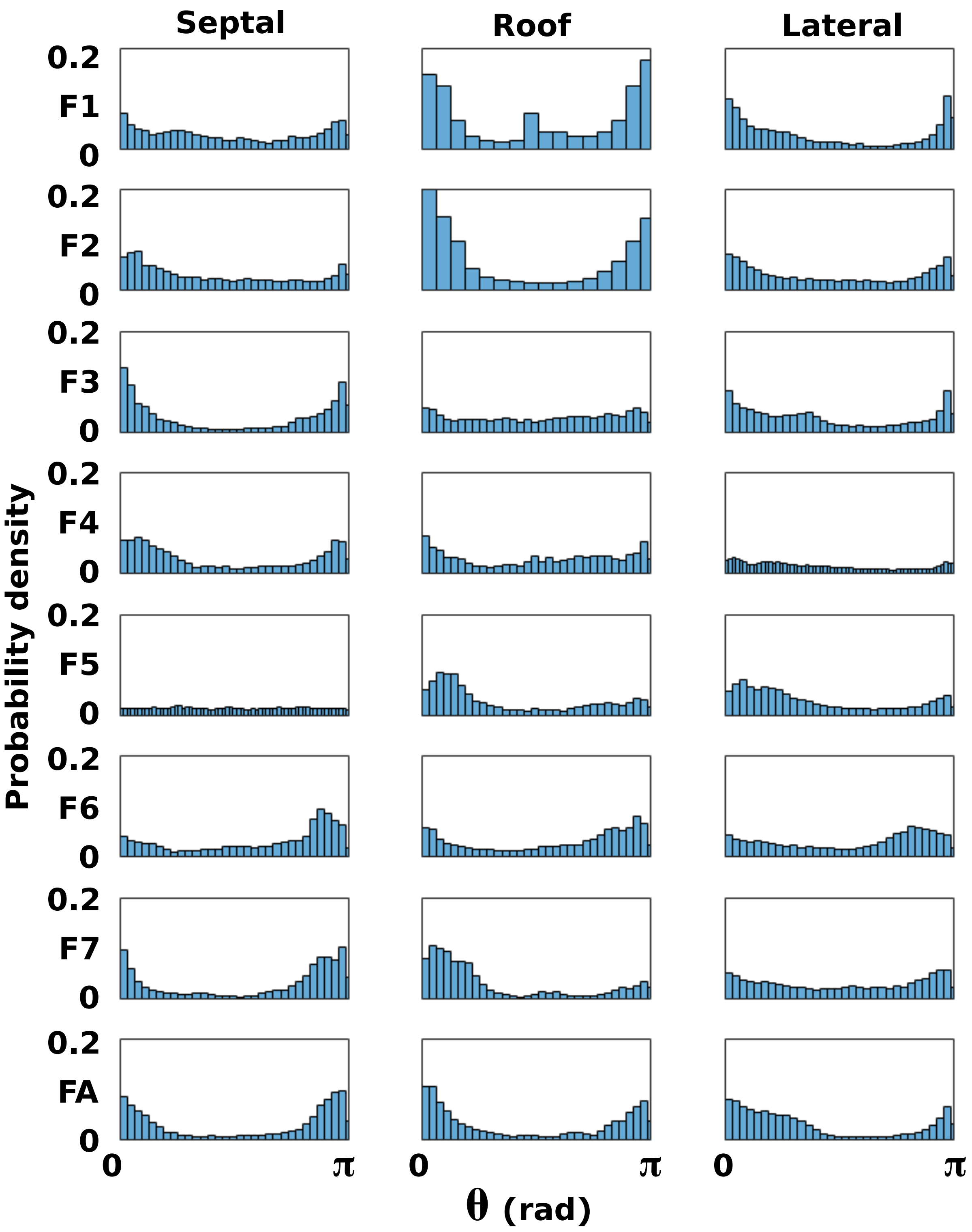

Figure 3: **UAC fibre angle distributions for the RA endocardium by atrial region.** Probability density histograms for each RA endocardial field for the lateral wall, roof and septal wall. Rows are for each fibre field 1-7 and the average field.

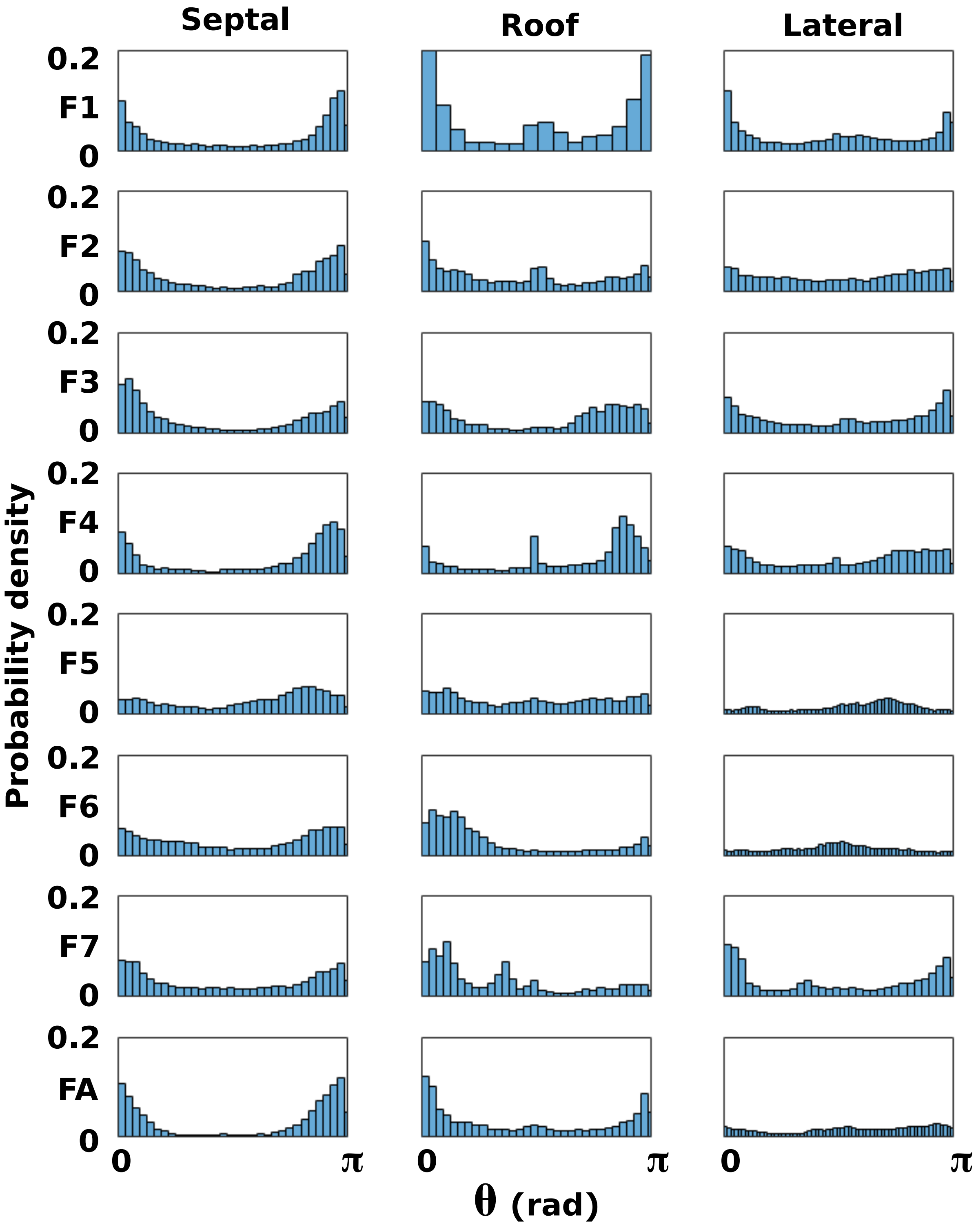

Figure 4: **UAC fibre angle distributions for the RA epicardium by atrial region.** Probability density histograms for each RA epicardial field for the lateral wall, roof and septal wall. Rows are for each fibre field 1-7 and the average field.

Table 1: LA endocardial and epicardial $Q_{\pi/8}$ values for different atrial regions. A1 - A7 compare the average fibre field to individual fibre fields 1-7 respectively. L1-L7 compare the Labarthe et al. fibre field to the individual fibre fields. AL is the average fibre field compared to the Labarthe et al. fibre field. Endo = endocardial; Epi = epicardial; Post = posterior; Ant = anterior; LAA = left atrial appendage; PV = pulmonary vein.

	A1	A2	A3	A4	A5	A6	A7	L1	L2	L3	L4	L5	L6	L7	AL
Endo Post	45.5	50.8	59.4	51.4	59.7	32.7	49.3	39.9	42.3	45.8	48.6	34.8	16.7	31.3	47.5
Endo Ant	47.7	36.7	53.8	60.5	46.2	45.4	50.6	28.5	44.6	28.4	26.6	30.9	27.8	30.0	32.4
Endo Roof	50.2	57.8	38.6	44.8	50.8	31.3	52.1	43.3	47.6	16.8	47.1	36.4	24.1	41.7	36.4
Endo LAA	37.3	51.3	44.9	52.7	40.7	43.5	46.2	26.4	20.9	25.3	25.7	28.6	23.9	27.2	27.2
Endo PV	38.6	46.3	43.5	45.8	46.9	37.9	47.6	30.0	25.6	24.8	33.7	34.6	27.8	25.0	28.9
Epi Post	42.0	41.0	56.3	47.3	42.2	50.8	42.6	29.7	32.9	37.9	33.4	36.5	33.8	33.3	48.5
Epi Ant	56.7	35.3	59.3	47.8	53.9	47.5	50.7	35.7	34.2	48.1	41.9	37.1	22.3	35.4	44.8
Epi Roof	47.4	40.3	40.4	46.8	43.6	50.7	48.2	29.7	28.5	27.1	31.6	30.3	30.0	27.8	25.6
Epi LAA	36.0	46.8	48.4	43.4	38.0	34.5	43.2	29.8	22.6	23.7	21.1	29.2	27.9	22.5	24.5
Epi PV	45.2	41.4	47.4	46.9	46.2	49.3	50.6	26.4	26.6	23.2	31.5	28.8	28.4	28.5	24.9

Table 2: RA endocardial and epicardial $Q_{\pi/8}$ values for different atrial regions. A1 - A7 compare the average fibre field to individual fibre fields 1-7 respectively. Endo = endocardial; Epi = epicardial; Sep= septal; Lat = lateral; RAA = right atrial appendage; VC =vena cavae.

	A1	A2	A3	A4	A5	A6	A7
Endo Sep	37.9	41.4	49.4	42.6	36.6	43.7	52.6
Endo Lat	61.8	47.1	50.5	41.3	59.1	43.6	52.0
Endo Roof	46.9	47.5	38.3	38.8	47.4	37.8	52.3
Endo RAA	52.9	31.7	46.3	48.9	35.5	42.5	48.8
Endo VC	45.5	41.6	40.9	40.7	35.9	38.7	31.6
Epi Sep	48.5	53.8	52.6	50.3	39.2	47.3	46.1
Epi Lat	53.2	46.4	50.2	55.1	47.8	41.5	50.2
Epi Roof	41.6	44.1	32.5	33.2	39.7	35.9	38.0
Epi RAA	41.4	49.4	35.8	44.7	40.6	47.6	46.0
Epi VC	42.2	43.9	41.9	41.8	41.5	39.8	38.1

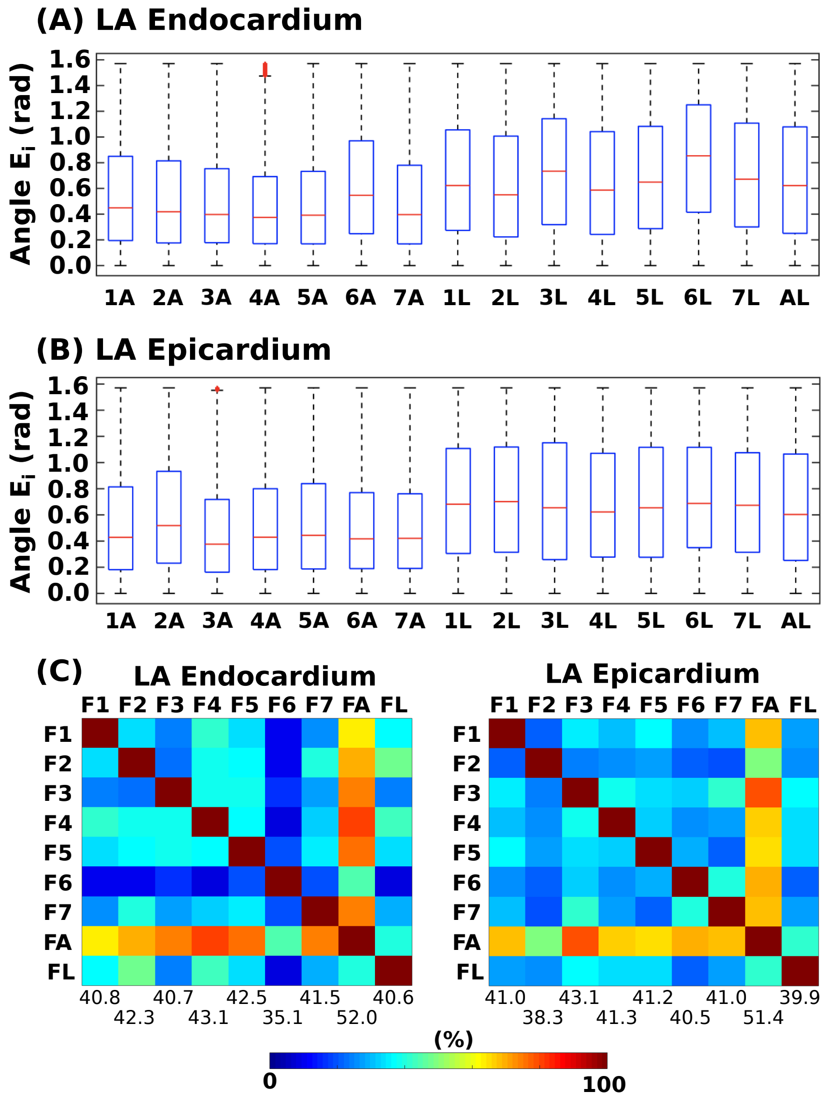

Figure 5: **Comparing individual left atrial fibre fields to the average fibre field and a rule-based fibre field.** (A) Box and whisker plots of angle E_i between fibre vectors for different LA endocardial maps. These were calculated between fibre fields 1-7 and the average map (labelled 1A - 7A), between fibre fields 1-7 and the Labarthe rule-based map (labelled 1L - 7L), and between the average field and the Labarthe rule-based map (AL). (B) Equivalent box and whisker plots for LA epicardial maps. (C) $Q_{\pi/8}$ between each pair of fibre fields calculated for the LA endocardium (left) and LA epicardium (right). Mean values calculated for each fibre field are given.

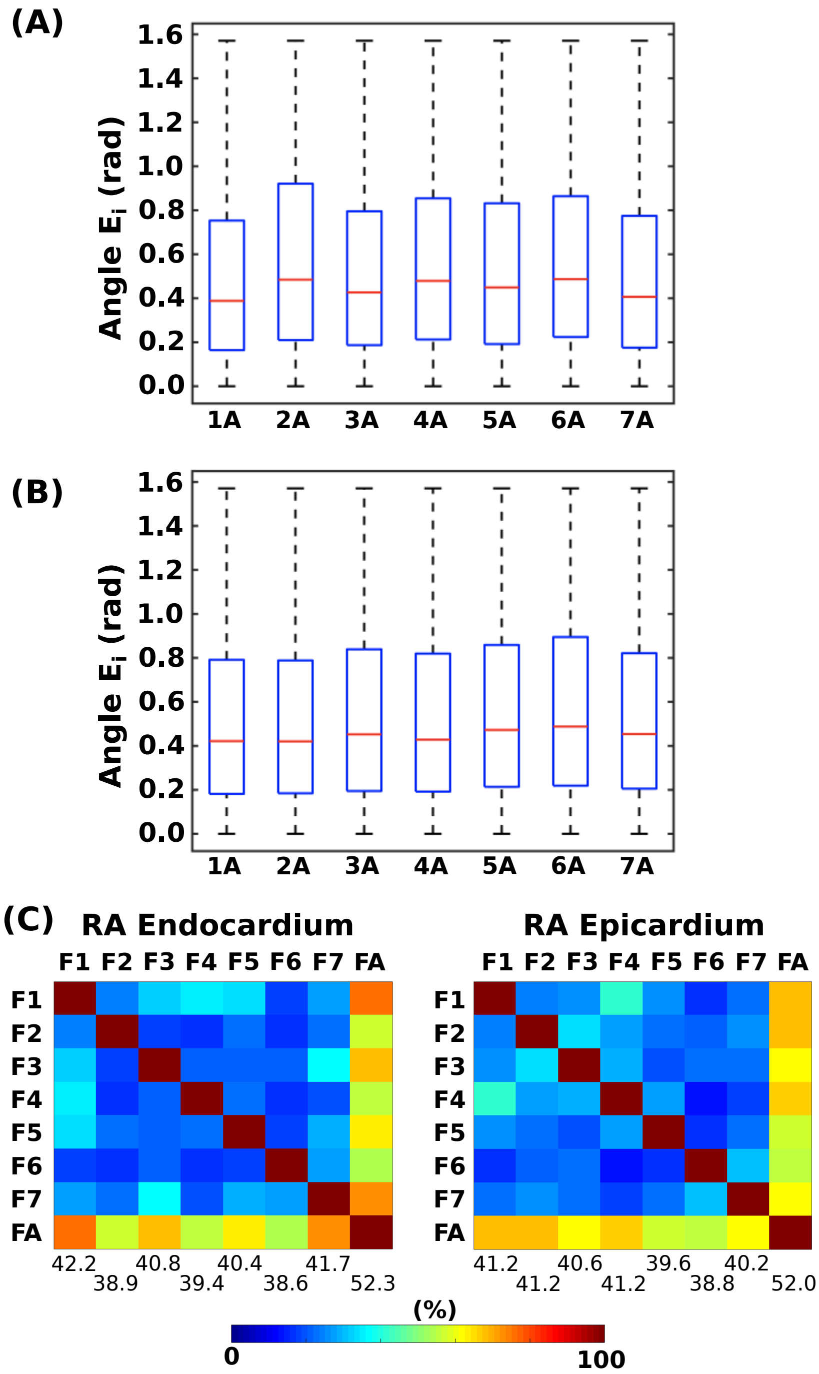

Figure 6: **Comparing individual right atrial fibre fields to the average fibre field.** (A) Box and whisker plots of angle E_i between fibre vectors for different RA endocardial maps. These were calculated between fibre fields 1-7 and the average map (labelled 1A - 7A). (B) Equivalent box and whisker plots for RA epicardial maps. (C) $Q_{\pi/8}$ between each pair of fibre fields calculated for the RA endocardium (left) and RA epicardium (right). Mean values calculated for each fibre field are given.

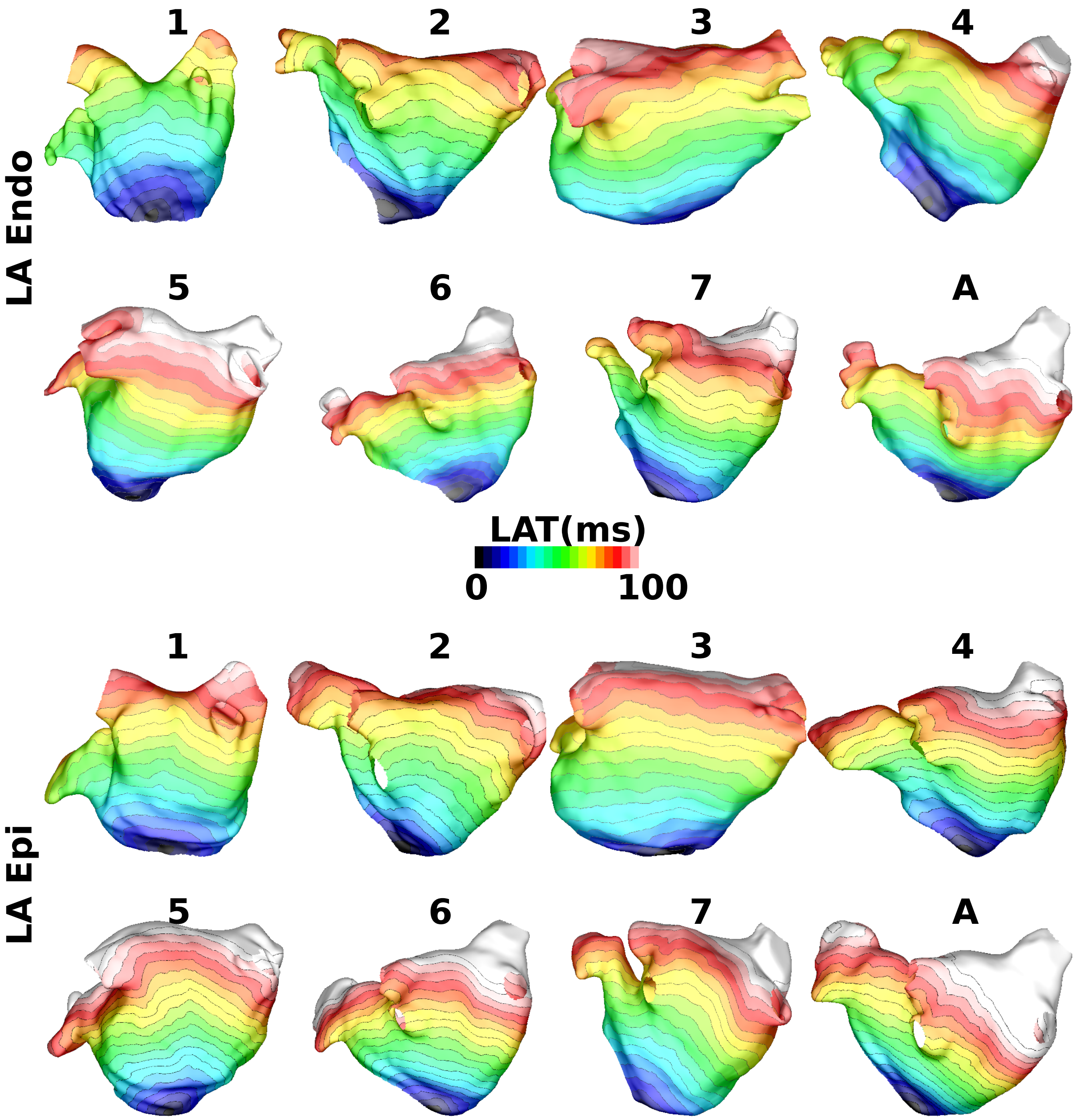

Figure 7: **Activation time maps for each LA fibre field.** Activation time maps for each of the fibre fields shown in Figure 4 of the main manuscript for LA endocardial and LA epicardial meshes and fibre fields. Pacing is from the coronary sinus and maps are in shown in posteroanterior view.

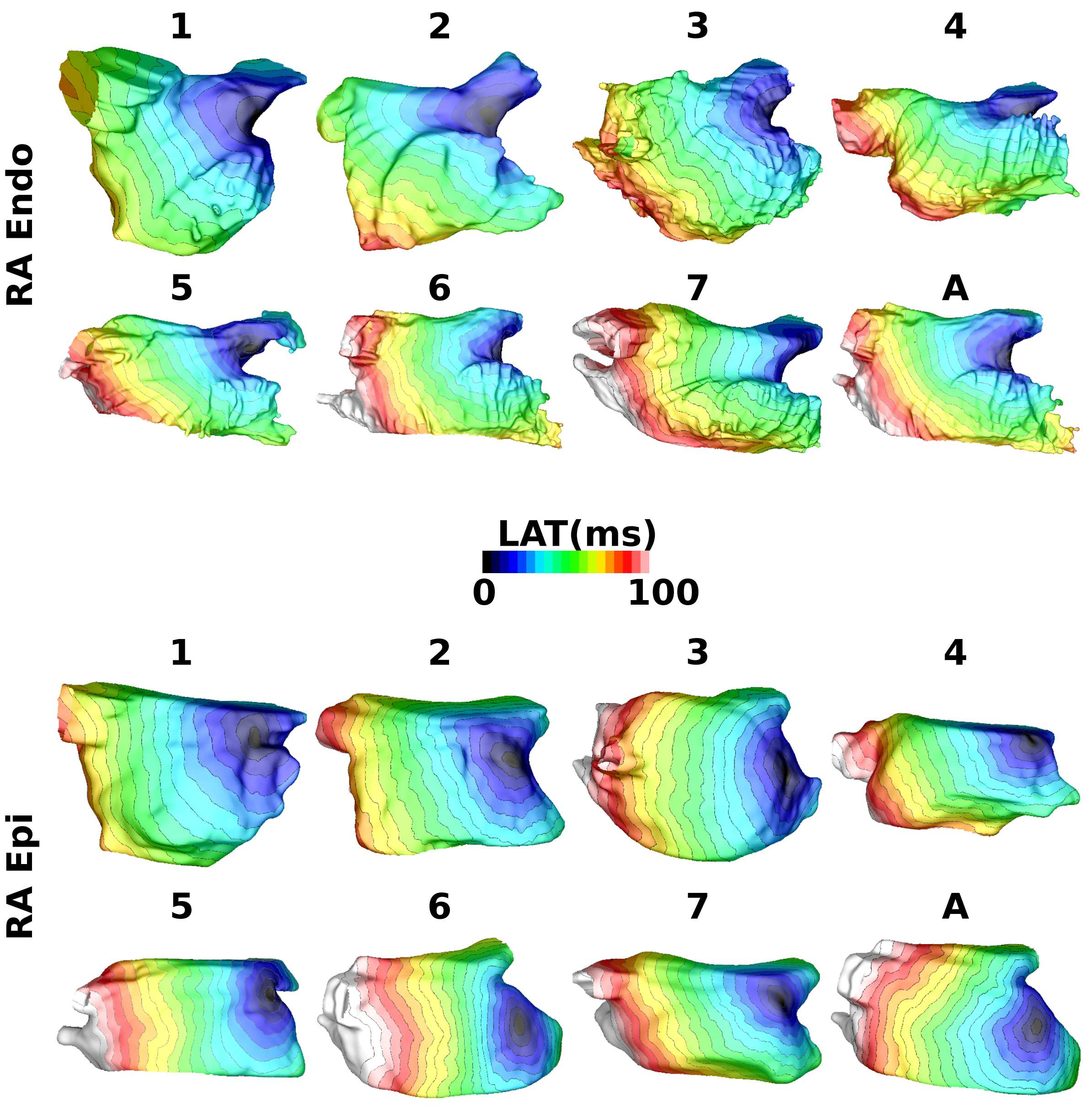

Figure 8: **Activation time maps for each RA fibre field.** Activation time maps for each of the fibre fields shown in Figure 5 of the main manuscript for RA endocardial and RA epicardial meshes and fibre fields. Pacing is from the SVC and maps are in shown in lateral-septal view.

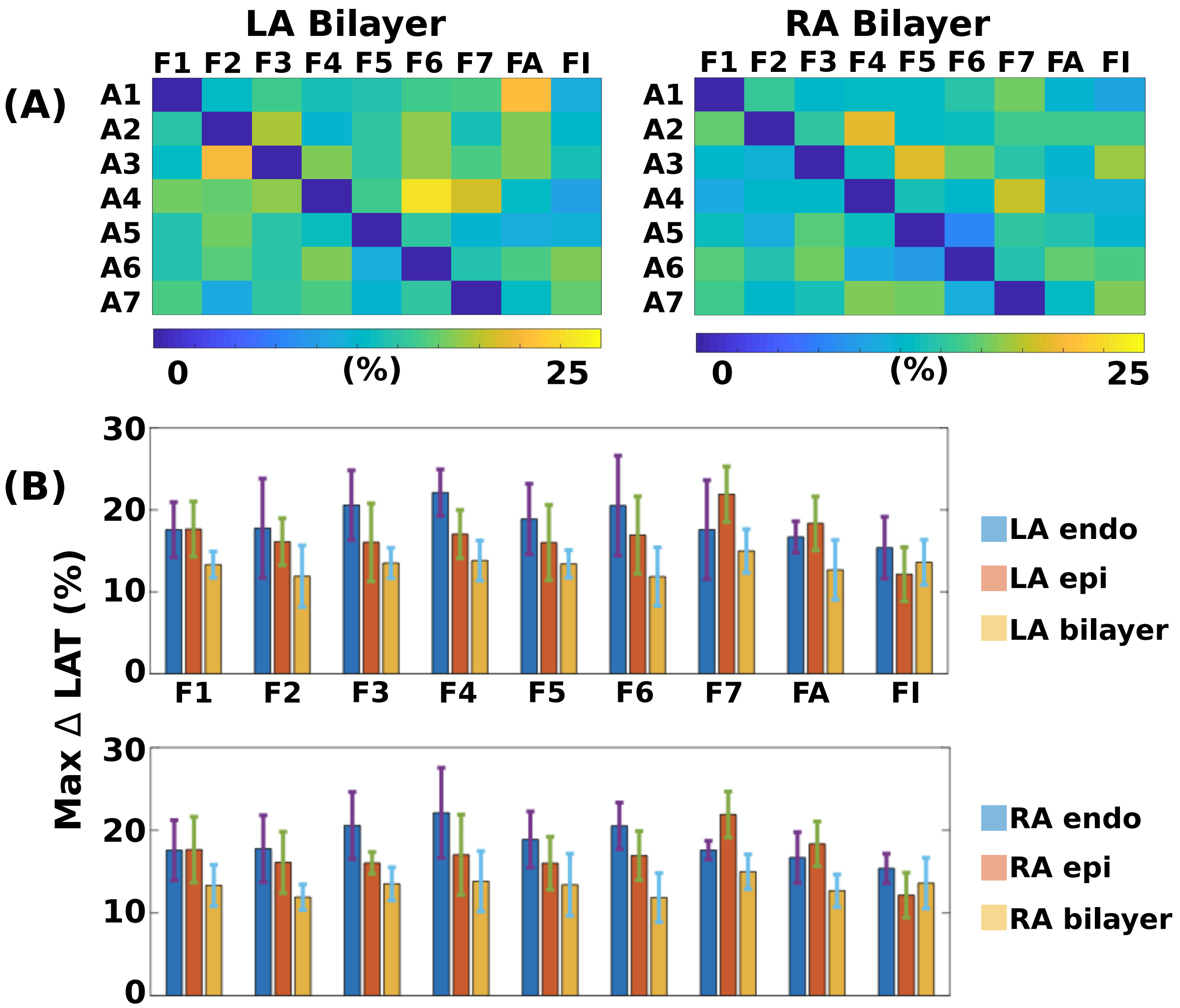

Figure 9: **Local activation time maps depend on fibre field.** (A) Maximum absolute LAT differences as a percentage of total activation time are shown for each anatomy (rows A1-A7) between each fibre field (columns F1-F7, FA and FI) and the gold standard LAT map (corresponding to the fibre field for that anatomy), for LA bilayer (left) and RA bilayer (right) simulations. (B) Bar charts showing the mean and standard deviation of the maximum absolute LAT differences as a percentage of total activation time for each fibre field (F1-F7, FA and FI), calculated across the 7 anatomies. These are given for the endocardial, epicardial and bilayer simulations for the LA (top) and RA (bottom). FA corresponds to the average fibre field; FI corresponds to the isotropic case.

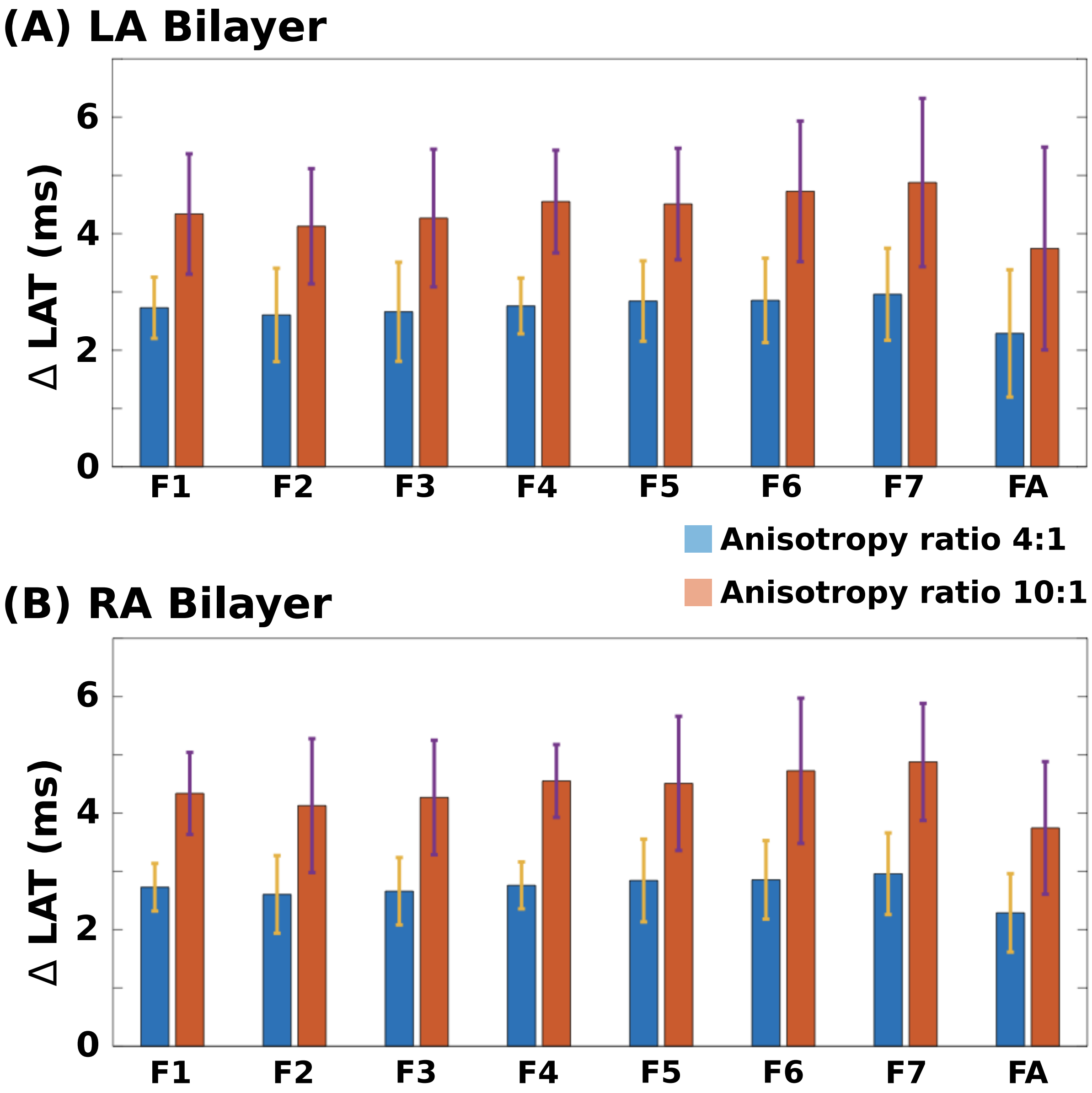

Figure 10: **Activation time maps depend on anisotropy ratio.** Increasing the anisotropy ratio from 4:1 to 10:1 increases LAT differences between different fibre field simulations. Bar charts showing the mean and standard deviation of the median absolute LAT differences for each fibre field (F1-F7 and FA), calculated across the 7 anatomies, for an anisotropy ratio of 4:1 (baseline, blue) and 10:1 (red). FA corresponds to the average fibre field. These are given for: (A) LA bilayer model and (B) RA bilayer model.

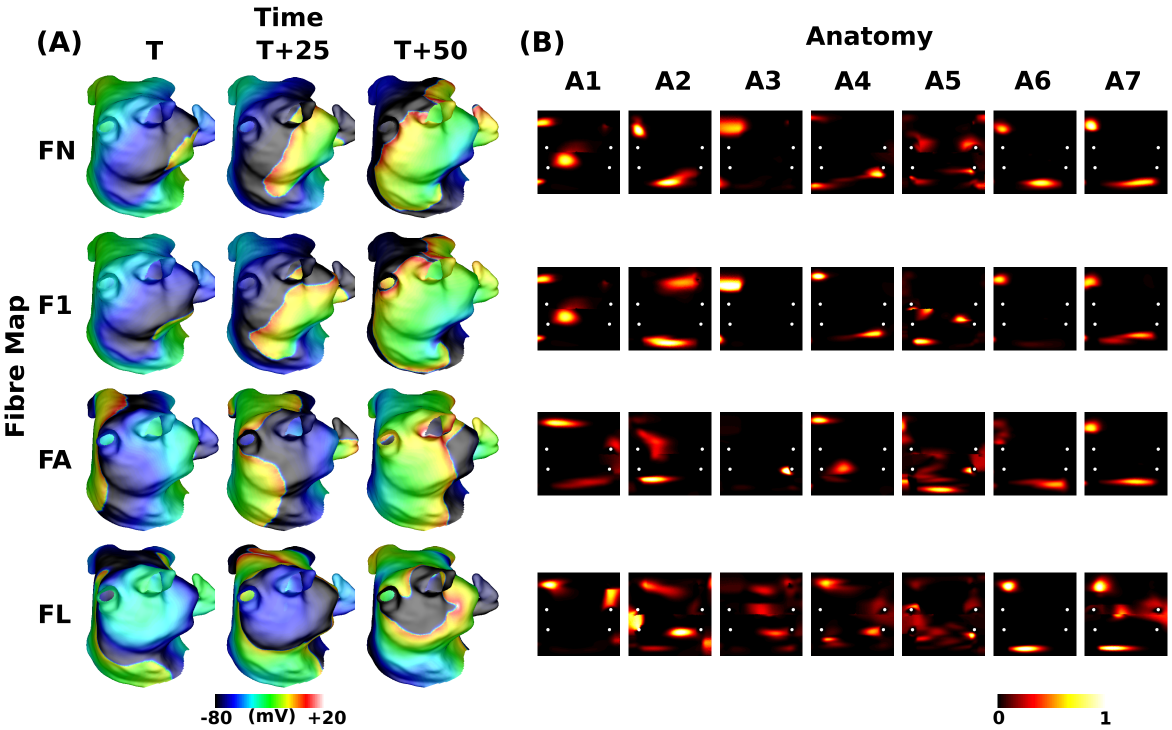

Figure 11: **Comparing simulations with the rule-based atlas of Labarthe et al[2] to simulations with DTMRI fibre fields.** (A) AF isopotential plots at 25ms intervals for anatomy 3 with fibre field 3 (denoted by FN as it is the fibre field corresponding to this anatomy, first row), fibre field 1 (F1, second row), the average fibre field (FA, third row), and the Labarthe atlas (FL, final row). Fibre field 1 exhibits a similar reentry to the original fibre field simulation (comparing the top two rows); whereas, the average fibre field and Labarthe rule-based atlas AF simulations are very different. Propagation is smoother for the rule-based Labarthe atlas (bottom row) to propagation for the other fibre fields. (B) Phase singularity density maps for the original fibre fields (denoted by FN, top row), fibre field 1 (F1, second row), the average fibre field (FA, third row), and the Labarthe fibre atlas (FL, bottom row), across anatomies A1-A7. Mean correlation coefficients are 0.44 for fibre field 1; 0.14-0.33 for fibre fields 2-7; 0.25 for the average fibre field; and 0.39 for the Labarthe et al[2] fibre atlas.
